# Reducing shoulder complaints in employees with high occupational shoulder exposures: study protocol for a cluster-randomised controlled study (The Shoulder-Café Study)

**DOI:** 10.1186/s13063-019-3703-y

**Published:** 2019-11-12

**Authors:** Jeanette Trøstrup, Lone Ramer Mikkelsen, Poul Frost, Annett Dalbøge, Mette Terp Høybye, Sven Dalgas Casper, Lene Bastrup Jørgensen, Thomas Martin Klebe, Susanne Wulff Svendsen

**Affiliations:** 1 Elective Surgery Centre, Silkeborg Regional Hospital, 8600 Silkeborg, Denmark; 2 Danish Ramazzini Centre, Department of Occupational Medicine, Regional Hospital West Jutland – University Research Clinic, Herning, Denmark; 3 Department of Clinical Medicine, Aarhus University, Aarhus, Denmark; 4 Danish Ramazzini Centre, Department of Occupational Medicine, Aarhus University Hospital, Aarhus, Denmark; 5 ErgoPro, Ry, Denmark

**Keywords:** Exercise, Intervention, Mechanical exposure, Occupation, Randomised controlled trial, Shoulder, Training programme

## Abstract

**Background:**

In Denmark, exercise therapy in combination with work modification is the first-choice treatment for persons with shoulder complaints and high occupational shoulder exposures. To obtain this treatment they must visit several healthcare providers, which makes usual care fragmented and uncoordinated. Therefore, we developed a new intervention which unifies the expertise that is needed. The main hypotheses are that a group-based Shoulder-Café intervention will more effectively reduce (1) shoulder complaints and (2) occupational shoulder exposures than an individual-based Shoulder-Guidance intervention (active control – enhanced usual care).

**Methods:**

A cluster-randomised trial is conducted including 120 employees with high occupational shoulder exposures. Companies (clusters) are randomised to either Shoulder-Café or Shoulder-Guidance with a 1:1 allocation ratio. Participants are 18–65 years old and have an Oxford Shoulder Score (OSS) ≤ 40. Both interventions include a home-based shoulder-exercise programme, assessment of shoulder exposures by technical measurements and self-report, and general information on how to reduce shoulder exposures. The Shoulder-Café course also includes three café meetings with physiotherapist-supervised exercises, clinical shoulder evaluation, education on shoulder anatomy, workplace-orientated counselling, and an opportunity for a workplace visit by a health and safety consultant. The primary outcomes are the OSS at 6-month follow-up (hypothesis I), and the mean number of min/day with the arm elevated > 60° shortly after the end of the intervention (hypothesis II). We will use a mixed-model analysis that allows for company clustering, and data will be analysed according to the intention-to-treat principle.

**Discussion:**

Persons with shoulder complaints and high occupational shoulder exposures are an obvious target group for secondary prevention efforts. We developed the Shoulder-Café to reduce shoulder complaints and shoulder exposures while unifying the expertise that is needed to evaluate and treat shoulder complaints. If the intervention is effective, it would warrant widespread implementation.

**Trial registration:**

Clinicaltrials.gov, ID: NCT03159910. Registered on 18 May 2017

## Background

Shoulder complaints prevail in the working-age population and constitute a common reason to consult a general practitioner [1]. In the general population, the prevalence of self-reported shoulder complaints is estimated to be 16–26% [1, 2] and in the general working population, the prevalence of subacromial impingement syndrome (SIS) has been reported to be 2–8% [3, 4]. In occupations with high mechanical shoulder exposures (work with elevated arms, repetitive shoulder movements, and forceful shoulder exertions), the risk of shoulder complaints and SIS is approximately doubled [5–10]. High occupational shoulder exposures are even associated with an approximately doubled risk of surgery for SIS [11–13], and when combined with shoulder complaints, a more than five-fold increase in risk of later surgery has been reported [14]. Based on these findings, persons with shoulder complaints and high occupational shoulder exposures seem an obvious target group for secondary prevention efforts.

The Danish Health Authority recommends exercise therapy as the first-choice treatment for shoulder complaints related to SIS [15, 16]. In case of shoulder complaints in combination with high occupational shoulder exposures, the Danish Health Authority also recommends work modifications [16]. Relevant modifications include reduction of exposures in specific job tasks (e.g. changes to work equipment and work practices, adjustments of workplace layout) and changes of the employee’s task distribution so that the duration of tasks with high exposures is reduced. To meet the recommendations of the Danish Health Authority, usual care today often entails repeated visits to several different healthcare providers (general practitioners, physiotherapists in private practice and municipalities, departments of orthopaedic surgery, departments of occupational medicine) and municipal job centres [17]. This makes usual care fragmented and uncoordinated as experienced by the patients [18]. To unify the necessary expertise to evaluate and treat shoulder complaints, a café intervention was recently developed and pilot-tested in Central Denmark Region [18]. The café concept was based on an intervention study of patients after lumbar spinal fusion, where participants in a Back-Café (three café meetings plus one exercise instruction by a physiotherapist, and featuring the opportunity to exchange experiences) scored better in daily function than participants in group-based physiotherapist-supervised exercises and individual-based video training [19]. This indicated the positive effects of a café concept per se. We further developed the pilot-tested café intervention [18] to target employees with shoulder complaints and high occupational shoulder exposures. Our café intervention, the Shoulder-Café, unifies clinical examination of the shoulders, patient education, supervised and home-based shoulder exercises, advice from a health and safety consultant on work modifications, and assessment of shoulder exposures at work.

Pain-related fear may be a reason why people avoid physical activities, including shoulder exercises, and reduction of an exaggerated reaction pattern of this kind might be part of the café intervention’s mechanism of action [20–22]. A Danish randomised controlled trial of the effectiveness of physical therapy exercises versus usual care after surgery for SIS showed that fear-avoidance beliefs (as measured by the Fear-Avoidance Beliefs Questionnaire – Physical Activity (FABQ-PA) scale in a version modified for the shoulder [23, 24] were reduced in the intervention group at 12-month follow-up (a reduction of 3 points was observed on a score ranging from 0 to 24 points with higher scores reflecting a higher tendency for fear-avoidance beliefs [25]). The same trial assessed Patients’ Global Impression of Change (PGIC) [26] and found that 65% of the patients in the exercise group experienced improvement in their shoulder condition compared to 49% in the usual care group [25]).

This trial compares a group-based Shoulder-Café intervention with an individual-based Shoulder-Guidance intervention (active control – enhanced usual care). The main hypotheses are that the Shoulder-Café will more effectively reduce (I) shoulder complaints and (II) occupational shoulder exposures than the Shoulder-Guidance. In relation to hypothesis I, we also expect a larger reduction of fear-avoidance beliefs, a larger improvement in PGIC, and larger improvements in a series of supplementary outcomes in the Shoulder-Café group than in the Shoulder-Guidance group.

## Methods

### Design and setting

The design is a cluster-randomised controlled trial with two parallel groups: Shoulder-Café and Shoulder-Guidance. We chose cluster-randomisation at the company level to prevent contamination between groups. T_0_ is the start of the intervention. With regard to hypothesis I, baseline data is collected shortly before T_0_ and follow-up data is collected by questionnaire 6 and 12 months after T_0_. With regard to hypothesis II, baseline data is collected shortly after T_0_ and follow-up data is collected shortly after end of intervention (EOI, around 3 months after T_0_). The setting is Central Denmark Region. A stakeholder group with members from trade unions, municipal rehabilitation centres, general practice, and the Health Planning Agency in Central Denmark Region has been established to facilitate the completion of the project and subsequent implementation of the Shoulder-Café if the results favour this intervention. This study protocol is written in accordance with the Standard Protocol Items: Recommendations for Interventional Trials (SPIRIT) Checklist [27] (Additional file 1 a and b) in conjunction with the Template for Intervention Description and Replication (TIDieR) Checklist [28].

### Trial population

The trial population consists of employees from occupations with high mechanical shoulder exposures who experience shoulder complaints. Relevant occupations are identified by means of a Danish Job Exposure Matrix (The Shoulder JEM), which is based on five experts’ ratings and covers all occupations in Denmark [29]. We selected occupations which fulfilled at least one of the following criteria: upper-arm elevation > 90° ≥ 1 h/day, highly repetitive work ≥ 0.5 h/day, moderately repetitive work ≥ 4 h/day, and a forceful shoulder exertion score ≥ 3 range (1 (light) to 5 (near maximal)) [11, 14]. Kitchen assistants with moderate exposures are also included to ensure sufficient representation of women. Companies are recruited in batches according to their geographical location. To achieve adequate patient enrolment, we will gradually widen the geographical distribution of companies within Central Denmark Region and include more occupational groups. The selected occupations are grouped according to industry: service (cleaning, kitchen and laundry assistants, hairdressers, and gardeners/paviours), manufacturing (dairy, bread, and wood-industry workers) and construction (electricians, carpenters, plumbers, bricklayers, house painters, welders, blacksmiths, and insulation workers). In a batch mode, we contact relevant companies in Central Denmark Region with at least 10 employees identified in The Central Business Register (https://datacvr.virk.dk/data/index.php?q=forside&language=en-gb). If a company accepts participation, employees from the relevant occupations are asked to fill in an electronic or postal screening questionnaire which – together with telephone screening – determines eligibility. The companies will distribute the questionnaires because, according to the Danish Data Protection Act, they are not allowed to give us a list with all possible participants. Thus, we cannot calculate the exact percentage that participated. We aim to include 120 participants in the trial (see the ‘Sample size’ section below).

**Fig. 1:**
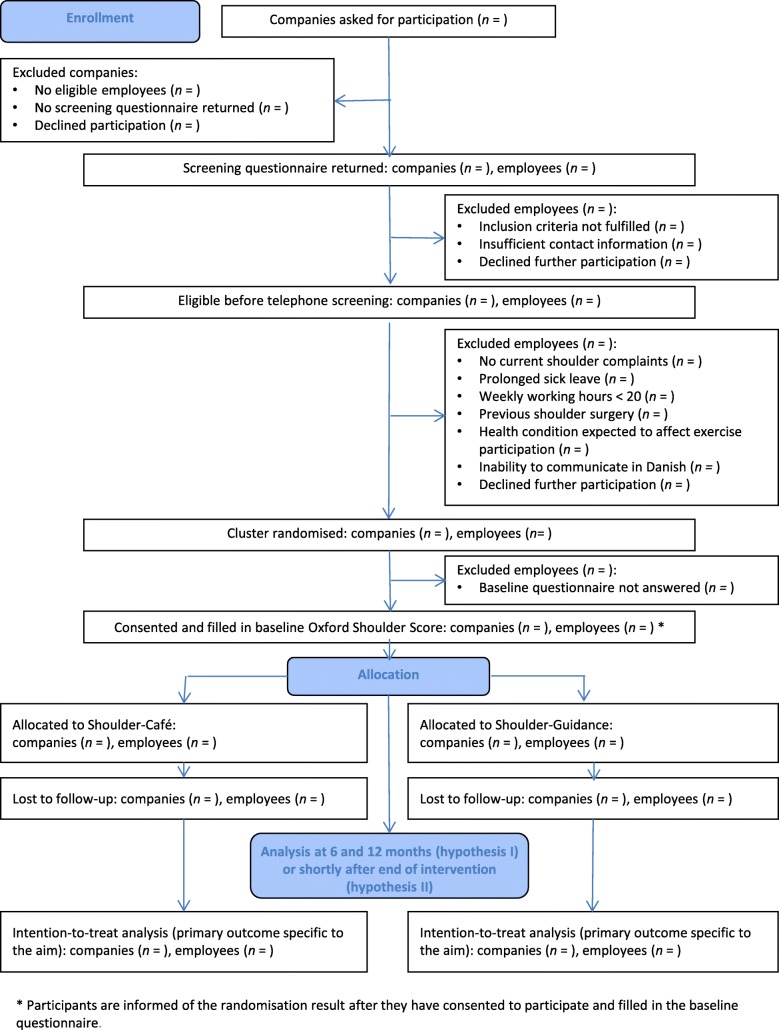
Expected flow of participants through the study

Based on the screening questionnaire, employees are invited to participate in the telephone screening if they meet the following inclusion criteria: aged 18–65 years, employed in one of the selected occupations, and with an Oxford Shoulder Score (OSS) ≤ 40 [30, 31]. The OSS, which exists in a Danish version [32], consists of 12 items, each referring to the past 4 weeks, with a total score ranging from 0 (worst) to 48 (best). We set the screening criterion at an OSS ≤ 40 to ensure that the included employees have shoulder complaints. The cut-off level was based on the pilot café intervention [18], where around 20% had an OSS ≤ 40, and is supported by mean scores of 42–47 in asymptomatic populations [33, 34]. Employees are excluded if they do not provide sufficient contact information or decline further participation. Based on the telephone screening, the following additional exclusion criteria are applied: no current shoulder complaints, sickness absence expected to continue into the intervention period, weekly working hours < 20, previous shoulder surgery, previous breast cancer operation, other health conditions expected to affect participation (e.g. rheumatoid arthritis, pregnancy), and inability to communicate in Danish. Employees may also decline further participation at this step. An additional exclusion criterion is failure to complete the baseline questionnaire (electronic or postal) before T_0_. The time between completion of the screening questionnaire and the telephone screening is expected to be around 5 weeks, and the subsequent time before enrolment is expected to be around 4 weeks. Companies are included if they are represented by at least one participant. Figure 1 presents the expected flow of participants through the study.

### Randomisation

Companies (clusters) are randomly allocated to Shoulder-Café or Shoulder-Guidance with a 1:1 allocation ratio using computer-generated random-number assignment. Randomisation is stratified by industry (service, manufacturing, construction) using blocking within strata with randomly permuted block sizes of 2, 4, and 6. A research assistant prepares closed envelopes with printed randomisation numbers and the corresponding intervention inside. Companies are contacted batch-wise. When all relevant employees from a company have completed screening, the principal investigator (JT) opens the envelope and invites eligible employees from the company to their first Shoulder-Café or Shoulder-Guidance attendance. The randomisation result is not revealed to the participants until they have signed the informed consent (obtained by JT) and completed the baseline questionnaire. The baseline questionnaire includes self-reported typical occupational shoulder exposures (see ‘Other assessments’ below), while baseline assessment of occupational shoulder exposures with respect to hypothesis II takes place after the randomisation result has been revealed.

### Interventions

The Shoulder-Café is designed as a complex intervention [35] with interacting components unified into a group intervention, whereas the Shoulder-Guidance is a simpler individual intervention. Consecutively, around 60 employees are scheduled to attend one of around 12 Shoulder-Café courses. Concurrently, around 60 employees are scheduled to attend a Shoulder-Guidance course. Each course lasts around 3 months with variations depending on practical issues; e.g. care givers’ time schedules. Physical attendance will take place at six geographically dispersed municipal health centres. A description of the Shoulder-Café and Shoulder-Guidance is presented in Table 1.

**Table 1:** Content and time schedule of the Shoulder-Café and the Shoulder-Guidance

Shoulder-Café	Shoulder-Guidance (active control – enhanced usual care)
1st café meeting (T_0_): • Distribution of home-based exercise pamphlet, BandCizer©, Axivity accelerometers^a^, diaries, andelastic bands • Presentation of participants and networking with the group • Supervised exercises with individual tailoring according to the exercise pamphlet • Clinical evaluation of the participants’ shoulders • Education about shoulder anatomy	1st intervention contact – individual appointment (T_0_): • Distribution of home-based exercise pamphlet, BandCizer©, Axivity accelerometers^a^, diaries, and elastic bands
At home: • Home-based exercises and exercise diary At work: • Shoulder exposure assessment and work diary	At home: • Home-based exercises and exercise diary At work: • Shoulder exposure assessment and work diary
2nd café meeting (~ 1.5 month after T_0_): • Written feedback on the 1st exposure assessment • Written general advice on reduction of occupational shoulder exposures • Supervised exercises with individual tailoring according to the pamphlet • Education about shoulder exposures • Advice on work modifications and possibility to ask questions about the 1st exposure assessment • Offer of a workplace visit to find ways to reduce the exposures • Networking with the group	2nd intervention contact – postal letter or email (~ 1.5 months after T_0_): • Written feedback on the 1st exposure assessment • Written general advice on reduction of occupational shoulder exposures
At home: • Home-based exercises and exercise diary	At home: • Home-based exercises and exercise diary
3rd café meeting (end of intervention ~ 3 months after T_0_): • Distribution^a^ of Axivity accelerometers and work diaries • Supervised exercises with individual tailoring according to the pamphlet • Networking with the group	3rd intervention contact – postal letter (end of intervention ~ 3 months after T_0_): • Distribution of Axivity accelerometers and work diaries
At work: • Shoulder exposure assessment and work diary	At work: • Shoulder exposure assessment and work diary
Postal letter or email: • Written feedback on the exposure assessment shortly after end of intervention	Postal letter or email: • Written feedback on the exposure assessment shortly after end of intervention
6-month follow-up (~ 6 months after T_0_): • Electronic or postal questionnaire	6-month follow-up (~ 6 months after T_0_): • Electronic or postal questionnaire
12-month follow-up (~ 12 months after T_0_): • Electronic or postal questionnaire	12-month follow-up (~ 12 months after T_0_): • Electronic or postal questionnaire

^a^The Axivity accelerometer is mounted, unless the participant is going on holiday or expects atypical work, e.g. due to course participation. A pamphlet "How to use Axivity" is handed out to all participants together with the accelerometer

The following elements are identical in the Shoulder-Café and the Shoulder-Guidance:

- A home-based shoulder-exercise programme with instructions for individual tailoring, described in a pamphlet (Additional file 2). Exercises for treating shoulder complaints have shown promising results [25, 36–38], but the optimal type, intensity, frequency, and duration of these exercises are not clear [39–43]. Our exercise programme was constructed by JT in cooperation with three physiotherapists from the Orthopaedic Shoulder Department at Silkeborg Regional Hospital (SRH). Based on studies showing the effect of exercise programmes [25, 36–38, 44], easily learned exercises were selected taking into account elements known to motivate exercise adherence (e.g. a limited number of exercises) [45]. The programme consists of four exercises: one posture-corrective exercise and three resistance exercises, performed bilaterally with an elastic band (Thera-band©). The three resistance exercises, each with three levels, consist of two exercises for the scapula-stabilising muscles (wall slide and low row/high row) and one for the rotator cuff muscles (external rotation). Participants are recommended to start with the exercises at level 1, and to perform three sets of up to 15 repetitions three to four times per week during the intervention period and preferably also thereafter. When a participant is able to perform three sets of 15 repetitions of an exercise without aggravating pain (lasting > 1 h after exercise), they are encouraged to progress to the next level of that particular exercise
- General information on occupational shoulder exposures and how to reduce them, described in a pamphlet (Additional file 3). The pamphlet, developed by AD, in collaboration with PF, SWS, and SDC, focusses on work with elevated arms, repetitive shoulder movements, and forceful shoulder exertions. It is based on previous assessments of occupational shoulder exposures [29], exposure-response relationships with shoulder disorders [11–14], and years of experience from work as occupational health physicians (PF and SWS) and as a health and safety consultant (SDC)
- Assessment of occupational shoulder exposures based on:
  - Technical measurements of postures and movements performed using an Axivity (AX3) accelerometer [46] processed to yield min/day with the arms elevated > 30°, > 60°, and > 90°, and median angular velocity (°/s) (as a measure of repetition) during work. Axivity measurements are performed on the more affected shoulder (right shoulder in case of similar symptoms). The accelerometer is fixed with double-sided adhesive tape to the lateral part of the upper arm with its proximal part just distal to the deltoid muscle. Data is recorded with a sampling rate of 50 Hz. The participants are instructed to wear the accelerometer for at least one and preferably five working days and to register working hours (start and stop times), main tasks, and whether it was a typical working day in a work diary. Data from one measurement day of ≥ 4 h per person is considered enough for characterisation at the group level
  - Self-reported estimates of the average level of forceful shoulder exertions for each working day using the Borg CR-10 scale [47]
  Exposure assessment is performed shortly after the first café meeting/intervention contact and shortly after EOI (see Table 1). All participants receive individual written feedback on their shoulder exposures after these two exposure assessment periods (Additional file 4).

#### Shoulder-Café

A Shoulder-Café course includes three café meetings spaced around 6 weeks apart. The principal investigator (JT) will attend all first and third café meetings. Each café meeting lasts for about 2 h and includes 15–30 min of ‘small talk’ and exchange of experiences over a cup of coffee/tea to secure social networking and interpersonal relationships. In addition, a Shoulder-Café course contains:

- Individually tailored shoulder exercises (in accordance with the exercise pamphlet, Additional file 2), supervised by physiotherapists from the six municipality health centres. At each café meeting, the attending physiotherapist spends 1 h demonstrating the exercises, correcting participants performing the exercises, and answering questions in relation to the exercises. To secure fidelity, the physiotherapists have attended a training session led by JT prior to the first café meeting and follow a pre-defined guideline (Additional file 5)
- A clinical shoulder evaluation of each participant performed at the first café meeting by a physiotherapist according to a pre-specified form (Additional file 6) and manual. The manual is based on the Danish guideline for diagnosing patients with shoulder complaints [15] and was developed by JT in cooperation with three physiotherapists from the Orthopaedic Shoulder Department at SRH, an orthopaedic surgeon (TK), and two occupational health physicians (PF and SWS). The aim of the examination is to characterise the participants clinically. If, as an exception, a participant is identified with a ‘red flag’ (e.g. progressive non-mechanical pain or weight loss) [48], they are advised to contact their general practitioner and a statement regarding advice against exercise is recorded; the participant will still be included in the intention-to-treat analyses. The three physiotherapists, who take turns performing the examinations, had been physiotherapists for 12–18 years, had special training in clinical evaluation of shoulder complaints, and had worked for 3–7 years in the Orthopaedic Shoulder Department at SRH at the start of the interventions
- Education about shoulder anatomy (Additional file 7) for 45 min at the first café meeting is provided by the above-mentioned experienced physiotherapists. The goal is to educate participants in the taking of appropriate action to reduce their shoulder complaints
- Workplace-orientated counselling focussing on reducing shoulder exposures. The counselling is given by a health and safety consultant (SDC), who had been a physiotherapist for 18 years and had been working as a health and safety consultant for 14 years at the start of the interventions. He has 45 min at his disposal at the second café meeting (Additional file 8), where he also answers questions about the individual feedback on shoulder exposures (Additional file 4). The counselling is based on theories from ‘The motivational conversation’ [49], ‘Stages of change’ [50], and ‘The health belief model’ [50] in order to increase the participants’ motivation for self-generated changes. There is also time to discuss organisational and other factors which might be barriers for work modifications. Previous experience indicates that health and safety advice is less likely to be implemented if the advice is too general or will take a long time to implement [51]. Therefore, our focus is on feasible and specific work modifications that can be implemented within a short time frame, i.e. modifications that are cheap, uncomplicated, and fit workplace conditions. Advice on more far-reaching modifications may also be given. A workplace visit by the health and safety consultant is an option when necessary to find ways to reduce the shoulder exposures. Plans of action that are based on a workplace visit are often focussed and clearly outlined, which increases their chances of being implemented [51]. The workplace visits are attended by the health and safety consultant, the participant, a working environment representative, and, if possible, the employer/supervisor. Initially, one to three tasks are prioritised. These entail high shoulder exposures and are difficult to perform while having shoulder complaints. Again, the focus is on specific work modifications that are feasible within a short time frame. The advice is documented in a workplace visit registration form by the health and safety consultant and categorised as ways to reduce high-task exposures (technical solutions) and ways to reduce the duration of tasks with high exposures (organisational solutions) for the individual participant. After the workplace visit, the health and safety consultant sends a summary of the advice to the employee, the working environment representative, and the employer/supervisor. We have resources for a maximum of 50 1-h workplace visits

The physiotherapists, who supervise the exercises and perform the clinical examinations, and the health and safety consultant are financially compensated by the project.

#### Shoulder-Guidance

The Shoulder-Guidance includes an initial 20–30-min individual appointment, staffed by a physiotherapist student or a project physiotherapist; the remaining parts of the guidance are delivered as postal letters or emails.

### Outcome measures

Additional file 11: Table S2 provides the time schedule of the trial and the timing of assessments of primary, secondary, and supplementary outcomes as well as assessments of baseline characteristics and measures of adherence and adverse events.

#### Primary outcomes

##### In relation to hypothesis I

The primary outcome is the OSS at 6-month follow-up. We chose a patient-reported outcome [52] which directly measures the participants’ shoulder complaints. The OSS has been translated and cross-culturally adapted to Danish [32] and is a valid, reliable, and responsive shoulder-specific measure [30, 53–56]. It is one of the recommended first-choice instruments in patients with shoulder disorders [57]. The OSS was developed for patients undergoing shoulder surgery [30], but has also been used in patients who have not been operated on [55, 56] and asymptomatic persons [33, 34]. Follow-up after 6 months was chosen to allow the potential effects on shoulder pain and disability to evolve.

##### In relation to hypothesis II

The primary outcome is work with the arm elevated > 60° (min/day) according to Axivity measurements shortly after EOI. This outcome was chosen based on the available evidence that work with elevated arms (assessed in various ways) is associated with an increased risk of shoulder complaints and SIS [5, 7, 8, 10] and because we think that this measure will be more responsive to change than min/day with the arm elevated > 90°, which has been quite well studied [10–14], but occurs to a limited extent in some of the included occupations. The timing was chosen because we expect that most work modifications will occur within the intervention period and because we want to use the second measurement feedback to motivate the participants for further work modifications.

#### Secondary outcomes

##### In relation to hypothesis I

Listed in order of priority, the secondary outcomes are:

- The OSS at 12-month follow-up. We added this time point because increasing effects of a training intervention 12 months after T_0_ has been reported previously [25]
- The FABQ-PA scale [23] at 6-month follow-up in a version modified for the shoulder [24]. The FABQ-PA scale contains four items about shoulder pain in relation to physical activity [20, 23, 24]. As mentioned in the ‘Background’ section, reduction of exaggerated fear-avoidance beliefs may be part of the café intervention’s mechanism of action [20–22]
- The PGIC [26] at 6-month follow-up, which reflects the participants’ general impression of change with regard to their shoulder condition rated on a 7-point Likert scale ranging from 1 (much better) to 7 (much worse) (https://www.sciencedirect.com/science/article/pii/S2287888215300684). Our a priori definition of improvement is the range 1 ‘Much better’, 2 ‘Better’, and 3 ‘A little better’
- The FABQ-PA scale [23] at 12-month follow-up

##### In relation to hypothesis II

Listed in order of priority, the secondary outcomes are:

- Min/day working with the arm elevated > 90° according to Axivity measurements shortly after EOI
- Mean median angular velocity (°/s) according to Axivity measurements shortly after EOI
- Average forceful shoulder exertions assessed by the Borg CR-10 scale [47] shortly after EOI
- Min/day working with the arm elevated > 30° according to Axivity measurements shortly after EOI

#### Supplementary outcomes

##### In relation to hypothesis I

Intensity of shoulder pain at rest and during activity measured on a numerical rating scale (NRS, ranging from 0 (no pain) to 10 (worst imaginable pain)), quick version of the Disabilities of the Arm, Shoulder and Hand (quick DASH) and work module [58], health-related quality of life using the EuroQol five-dimension, three-level health survey (EQ 5D-3 L) [59], work ability using the Work Ability Score [60, 61], PGIC at 12 months’ follow-up, overall satisfaction with the intervention at 6 and 12 months, and the degree to which the participant felt sufficiently informed about (1) how to handle shoulder complaints, (2) how to perform shoulder exercises, and (3) how to reduce occupational shoulder exposures at 6-month follow-up (5-point scales).

##### In relation to hypothesis II

Work modifications according to questionnaire information at 6-month follow-up.

Supplementary outcome measures will be selected from these variables.

### Other assessments

Other baseline assessments are smoking status, body mass index, duration of shoulder complaints, psychosocial work exposures (job demands, job control, and social support based on the Karasek-Theorell model) [62], occupational mechanical shoulder exposures (self-reported upper-arm elevation, repetitive shoulder movements, forceful shoulder exertions, and use of vibrating tools). In addition, job title, weekly working hours, and system of wage payment are assessed at baseline and at 12-month follow-up and work status is assessed at 12-month follow-up. At 6- and 12-month follow-up, all participants are also asked how often exercise was performed.

### Adherence

Adherence to the home-based exercise programme is monitored using an exercise diary and a BandCizer© sensor mounted on the elastic band (Thera-band©). The BandCizer© records the exercise-dose quantified as time under tension [63–65]. Adherence to the exposure assessment will be described as the percentage of the participants who have one work day or more with ≥ 4 h of Axivity data and/or a Borg CR-10 rating in the first and in the second exposure assessment period. For the Shoulder-Café group, adherence to café meetings will also be described (Additional file 11: Table S2).

### Co-interventions and adverse events

The questionnaires at 6- and 12-month follow-up will ask about co-interventions and adverse events (Additional file 11: Table S2).

### Data collection and data management

All questionnaires will be collected by the principal investigator (JT). Companies will be reminded by email and telephone if few or no screening questionnaires have been returned after 1–2 months. Participants who do not return the follow-up questionnaires will be reminded to do so by email and finally by postal letter. Data from the paper screening questionnaires will be scanned by PostNord [66]. Data from electronic screening, baseline, and follow-up questionnaires will be directly captured in REDCap (version 7.4.17, Vanderbilt University), while data from the paper versions of the baseline and follow-up questionnaires and from exercise diaries will be manually entered into REDCap. Data from the BandCizer© will be processed to yield date, number of training sessions, number of exercise sets, number of repetitions, time under tension for each repetition, and total time under tension for each training session. Variables based on data from the BandCizer© will be entered into REDCap. Axivity data (Axivity Ltd., Newcastle upon Tyne, United Kingdom) will be downloaded using OmGui open-source software (OmGui Version 1.0.0.28; Open Movement, Newcastle University, Newcastle upon Tyne, United Kingdom) and saved in raw format files. MatLab (Build 8.6.0.267246 (R2015b) 64 bit) and STATA 15 (StataCorp LP, College Station, TX, USA) will be used for data processing and statistical analyses. Data cleaning will be documented in Stata do files. Questionnaires and other documents, which are not provided as supplementary materials (Additional files 1, 2, 3, 4, 5, 6, 7, 8, and 9), are available in Danish and can be requested from JT (Additional file 10).

### Blinding

Blinding of participants and care providers is not possible due to the character of the interventions. To prevent this from influencing the answers on the OSS and other patient-reported outcomes, all participants receive an active intervention. With respect to shoulder exposures, the outcome assessor (AD) will be blinded to intervention arm. We have developed a statistical analysis plan (SAP) to minimise the risk of analysis bias (Additional file 9).

### Sample size

We aim to be able to show a minimum clinically important difference between the groups of at least 5 points in the OSS [67, 68] at 6-month follow-up. With an expected SD of 8 points [25], an intraclass correlation coefficient of 0.05 [69, 70], and a mean cluster-size of four, the study size needs to be ≥ 96 (2 × 48) with a two-sided significance level of 0.05 and a power of 0.80. We aim to include 60 employees in each group to ensure that 50 employees in each group complete the study. Power calculations were carried out with Stata 15 (StataCorp LP, College Station, TX, USA; power twomeans with cluster option).

### Statistical methods

All analyses will be performed according to intention-to-treat principle. Regarding hypothesis I, a mixed-model analysis of the OSS will be performed including ‘intervention’ (Shoulder-Café and Shoulder-Guidance), ‘time’ (6- and 12-month follow-up), ‘intervention × time’, baseline OSS, sex, age, and industry (service, manufacturing, construction) as fixed effects, adjusting for random effects of participant and company (cluster). The FABQ-PA will be analysed likewise, but will be adjusted for baseline FABQ-PA instead of baseline OSS. In the analysis of PGIC at 6 months the outcome will be dichotomised as described above. We will use a risk-difference model if around 50% of the participants improve. If a considerably smaller percentage (< 20%) improves, we will employ a relative-risk model using improved as the outcome, while, if a considerably larger percentage (> 80%) improves, we will employ a relative-risk model using ‘not improved’ as the outcome. The analysis of PGIC will be adjusted for sex, age, and industry and use robust standard errors to take clustering at company level into account.

Regarding hypothesis II, a mixed-model analysis of the primary outcome (min/day working with the arm elevated > 60°) will be performed including ‘intervention’ (Shoulder-Café and Shoulder-Guidance), baseline min/day working with the arm elevated > 60°, sex, age, and industry (service, manufacturing, construction) as fixed effects, adjusting for random effects of company (cluster). The analyses for the secondary outcomes will be performed likewise, but will be adjusted for the respective baseline values instead of the baseline number of min/day working with the arm elevated > 60°.

If no more than two questions in the OSS are left unanswered, single mean imputation will be used [31], otherwise the total score will be left missing. Axivity measures are considered missing in case of < 4 h of measurement data during one working day. Loss to follow-up will be addressed by sensitivity analyses comparing realistic scenarios; subgroup analyses are not intended. Additional information is available in the SAP (Additional file 9).

### Harms and data monitoring

The intervention is based on non-invasive methods and is not expected to cause any adverse events other than possible temporary muscle tenderness after shoulder exercises. Therefore, no data monitoring committee has been established and no stopping rules defined. Any unexpected serious adverse event will be reported to the Committee on Health Research Ethics in Central Denmark Region within 7 days after the principal investigator (JT) has become aware of the event.

### Publication policy

Hypotheses 1 and 2 will be addressed in separate publications. The main publication regarding hypothesis I will be prepared first and the main publication regarding hypothesis II shortly thereafter. We intend to publish positive, negative, and inconclusive results. Authorship will be determined in accordance with the recommendations of the International Committee of Medical Journal Editors. Furthermore, we plan to disseminate the results to key stakeholders through the projects’ stakeholder group. The authors do not have any publication restrictions.

### Satellite studies

Two prospective cohort studies are planned based on the cluster-randomised trial. One study, with the OSS as the primary outcome, will investigate the relative influence of shoulder exercises and reduced occupational shoulder exposures on shoulder complaints. Another study will investigate the intensity of shoulder pain at rest and during activity (NRS) monitored week by week using short message service as a predictor of subsequent weekly exercise dose, and the potential influence of fear-avoidance beliefs on this relationship. Further, a process evaluation [71, 72] is nested in the trial to assist later contextualisation of the outcomes. The findings from this may point to areas that warrant further consideration or development prior to a potential wider implementation of the Shoulder-Café intervention. The process evaluation employs semi-structured interviews [73] with eight participants from the Shoulder-Café (*n* = 4) and Shoulder-Guidance (*n* = 4) conducted 1 month after EOI and 12 observations [74] of Shoulder-Café (*n* = 9) and Shoulder-Guidance (*n* = 3) sessions. All interviews and observations are supervised by a senior project participant (MTH). Further, a focus group interview is conducted with self-selected professionals (physiotherapists from hospital and municipalities and the health and safety consultant) (*n* = 12).

## Discussion

Several studies have found that exercise is effective in reducing shoulder complaints [25, 36–41, 43, 75, 76], but optimal ways to exercise remain to be established. Few studies have evaluated interventions that have addressed occupational shoulder exposures in order to prevent or reduce shoulder complaints [77–79]. The disappointing results of these studies may be related to the fact that for the most part they were completed in office environments and healthcare settings, where shoulder exposures are at most moderate to begin with [77–79]. Only one study that we are aware of included participants with high shoulder exposures, but did not document whether the intervention reduced the exposures [80]. The combination of shoulder exercises and workplace-orientated advice using a café concept is a novel approach, which minimises the fragmentation that is characteristic of usual care today and adds potential benefits of delivering the intervention in a group setting rather than individually [81] (e.g. social support in combination with professional guidance and exchange of ideas for improving work practices between group members).

The strengths of this study are the randomised controlled design, cluster-randomisation at company level to prevent contamination between groups, use of validated patient-reported outcomes to assess shoulder complaints, and technical measurements of shoulder postures and movements.

Stigmatisation of employees with shoulder complaints is avoided as the intervention takes place outside the company and after working hours. This enables participants to decide whether they want to inform their workplace about their participation.

A limitation of the study is the inability to blind participants to the intervention, but both groups receive an active intervention in order to reduce the risk of biased outcome reporting. Baseline assessment of occupational shoulder exposures takes place after the randomisation result has been revealed. However, Axivity accelerometers are mounted on all participants at their first intervention appointment and we use technical measurements performed on several working days. This should guard against differential participation and differential misclassification of occupational shoulder exposures. Additionally, participants and non-participants will be compared with respect to self-reported occupational shoulder exposures according to the baseline questionnaire. To minimise the risk of analysis bias, we have developed a SAP prior to any analysis.

A further limitation is that it is not possible to differentiate between the separate effects of exercise, work modification, diagnostic clarification, education, workplace-orientated counselling, and group processes on the participants’ shoulder complaints, but the analyses in relation to hypothesis II and one of the planned satellite studies will reveal to which extent reduced occupational shoulder exposures may have played a part. To give a further indication of the relative influence of the intervention elements, we will ask the participants at 6-month follow-up to which degree they feel that the intervention provided them with sufficient knowledge about (1) how to handle shoulder complaints, (2) how to exercise, and (3) how to reduce their shoulder exposures. The process evaluation may aid in this evaluation. If shoulder exposures are reduced by handing over high-load tasks to colleagues, the problem may only be relocated. On the other hand, the possibility of exposure modification in periods with increased pain may be in all employees’ favour.

If the results turn out to be positive, we believe that the Shoulder-Café intervention has the potential to be implemented on a larger scale. The pilot-tested café intervention is already implemented in three municipalities in Central Denmark Region, and the project has a stakeholder group to back up the process. Further, it should be possible to develop the intervention to involve other musculoskeletal regions, which has already been requested by one of the participating municipalities.

### Trial status

Protocol version 1.0: Issue date: 22 January 2019. Recruitment of participants started in May 2017 and is ongoing. Recruitment of participants is expected to end no later than June 2019.

## Supplementary information

**Additional file 1.** a: Standard Protocol Items: Recommendations for Interventional Trials (SPIRIT) Checklist. b: World Health Organisation (WHO) Trial Registration Data Set.

**Additional file 2.** Pamphlet – Home-based shoulder exercise programme. (Images on page 1 were bought from Colorbox. Other photos are our own).

**Additional file 3.** Pamphlet – How to reduce occupational shoulder exposures. (Images are our own).

**Additional file 4.** Individual feedback on occupational shoulder exposures.

**Additional file 5.** Guideline for supervised exercises.

**Additional file 6.** Clinical shoulder examination form.

**Additional file 7.** Educational slides – shoulder anatomy. (Images on page 3 were bought from Colorbox. Other photos are our own).

**Additional file 8.** Educational slides – workplace counselling. (Images are our own).

**Additional file 9.** Statistical analysis plan (SAP).

**Additional file 10.** List of questionnaires and other documents used in the project.

**Additional file 11:** **Table S2.** Schedule for study procedures. For each batch of companies, the two interventions (Shoulder-Café and Shoulder-Guidance) start and end simultaneously.

## Data Availability

Not applicable since this manuscript is a study protocol.

## Abbreviations

CI: Confidence interval
EOI: End of intervention
FABQ-PA: Fear Avoidance Beliefs Questionnaire – Physical Activity
OSS: Oxford Shoulder Score
PGIC: Patients’ Global Impression of Change
SAP: Statistical analysis plan
SRH: Silkeborg Regional Hospital
T_0_: Start of intervention
