# Supplementary material for: Reducing shoulder complaints in employees with high occupational shoulder exposures: study protocol for a cluster-randomised controlled study (The Shoulder-Café Study)
Source: Trials. 2019 Nov 12;20:627. doi: 10.1186/s13063-019-3703-y (PMC6852773; doi:10.1186/s13063-019-3703-y)
Supplement: Supplementary file 1 — Additional file 1. a: Standard Protocol Items: Recommendations for Interventional Trials (SPIRIT) Checklist. b: World Health Organisation (WHO) Trial Registration Data Set. [file 13063_2019_3703_MOESM1_ESM.zip › Additional file 1b WHO Trial Registration Data SetR1.pdf]

Additional file 1b: WHO Trial Registration Data Set

1. Primary Registry and Trial Identifying Number	Clinicaltrials.gov: NCT03159910
2. Date of Registration in Primary Registry	18 May 2017
3. Secondary identifying Numbers	The Danish Data Protection Agency (1-16-02-498-16); The Committee on Health Research Ethics in Central Denmark Region (1-10-72-271-16)
4. Source(s) of Monetary or Material Support	Central Denmark Region (Folkesundhed i Midten) (A911); Danish Regions and the Danish Health Confederation (Sundhedskartellet, udviklings- og forskningspuljen); Task Force for Health Innovation, Central Denmark Region; Aarhus University; The Association of Danish Physiotherapists' fund for research; The Danish Rheumatism Association (A5659); Sawmill owner Jeppe Juhl and wife Ovita Juhls Memorial trust (for SWS' research), and Helga og Peter Korning's Fund
5. Primary Sponsor	Regional Hospital Central Jutland
6. Secondary Sponsor(s)	Regional Hospital West Jutland; Aarhus University Hospital; Aarhus University
7. Contact for Public Queries	Jeatro@rm.dk, 0045-24759153, Elective Surgery Centre, Silkeborg Regional Hospital, 8600 Silkeborg, Denmark
8. Contact for Scientific Queries	Jeanette Trøstrup MsC, jeatro@rm.dk, 0045-78415000, Elective Surgery Centre, Silkeborg Regional Hospital, 8600 Silkeborg, Denmark
9. Public Title	Reducing shoulder complaints and shoulder exposures
10. Scientific Title	Reducing shoulder complaints in employees with high occupational shoulder exposures: a cluster-randomised controlled study (The Shoulder-Café Study)
11. Country of Recruitment	Denmark
12. Health Condition / Problem Studied	Shoulder pain; occupational shoulder exposures
13. Intervention(s)	Shoulder-Café<br><br>Shoulder-Guidance (active control – enhanced usual care)
14. Key Inclusion and exclusion Criteria	*Inclusion criteria*: 18–65 years, employed in occupations with high mechanical shoulder exposures (service, manufacture, construction), shoulder complaints<br><br>*Exclusion criteria*: Previous shoulder surgery, health condition expected to affect participation, sickness absence expected to continue into the intervention period, inability to communicate in Danish
15. Study Type	Cluster-randomised controlled study. Companies (clusters) are randomly allocated to Shoulder-Café or Shoulder-Guidance

	with a 1:1 allocation ratio. Randomisation is stratified by industry using blocks within strata with randomly permuted block sizes of 2, 4, and 6<br><br>Blinding of participants and care providers is not possible due to the character of the interventions<br><br>Purpose: To investigate if a group-based Shoulder-Café will reduce (I) shoulder complaints and (II) occupational shoulder exposures more effectively than an individual-based Shoulder-Guidance intervention
16. Date of First Enrollment	August 2017
17. Sample Size	Plan to enrol: 120 participants
18. Recruitment Status	Recruiting: participants are currently being recruited and enrolled
19. Primary Outcome	I. Shoulder complaints: the Oxford Shoulder Score. Method: Danish version (questionnaire). Time point: Follow-up 6 months after start of intervention (T_0)<br>____________________<br>II. Occupational shoulder exposures: work with elevated arms > 60˚ (minutes/day). Method: Axivity accelerometer. Time point: Follow-up shortly after end of intervention (EOI, around 3 months after T_0)
20. Secondary Outcomes	Ia. The Oxford Shoulder Score. Method: Danish version (questionnaire). Time point: Follow-up 12 months after T_0<br><br>Ib. Fear Avoidance Beliefs Questionnaire – physical activity scale (FABQ-PA). Monitored with a questionnaire version modified to the shoulder. Time point: Follow-up 6 and 12 months after T_0<br><br>Ic. Patients' global impression of change. Method: Questionnaire - 7 point Likert scale. Time point: Follow-up 6 and 12 months after T_0<br>____________________<br>IIa. Work with elevated arms > 90˚ (minutes/day). Method: Axivity accelerometer. Time point: Follow-up shortly after EOI<br><br>IIb. Repetitive shoulder movements (mean median angular velocity (˚/s)). Method: Axivity accelerometer. Time point: Follow-up shortly after EOI<br><br>IIc. Work with forceful shoulder exertions: Borg CR-10 scale. Method: Work diary. Time point: Follow-up shortly after EOI<br><br>IId. Work with elevated arms > 30˚ (minutes/day). Method: Axivity accelerometer. Time point: Follow-up shortly after

	EOI
21. Ethics Review	The Committee on Health Research Ethics in Central Denmark Region approved the trial on 20 March 2017 (1-10-72-271-16).<br>Contact: Regionssekretariatet - Region Midtjylland, Skottenborg 26,8800 Viborg, Denmark.<br>Tel. +45 7841 0183, email: komite@rm.dk
22. Completion date	Estimated in December 2020
23. Summary results	Please, see the study protocol including the statistical analysis plan
24. IPD Sharing statement	Undecided
