# Supplementary material for: Reducing shoulder complaints in employees with high occupational shoulder exposures: study protocol for a cluster-randomised controlled study (The Shoulder-Café Study)
Source: Trials. 2019 Nov 12;20:627. doi: 10.1186/s13063-019-3703-y (PMC6852773; doi:10.1186/s13063-019-3703-y)
Supplement: Supplementary file 2 — Additional file 2. Pamphlet – Home-based shoulder exercise programme. (Images on page 1 were bought from Colorbox. Other photos are our own). [file 13063_2019_3703_MOESM2_ESM.pdf]

Additional file 2: Home-based shoulder exercise programme (Images side 1 is bought from Colorbox)

ID number: ___ ___ ___ ___

Participant name: ____

Home-based shoulder exercise programme

Reducing shoulder complaints and shoulder exposures

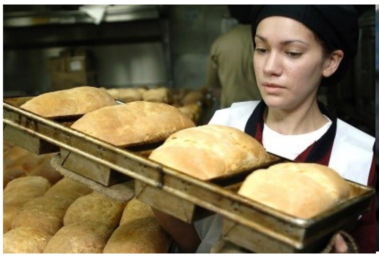
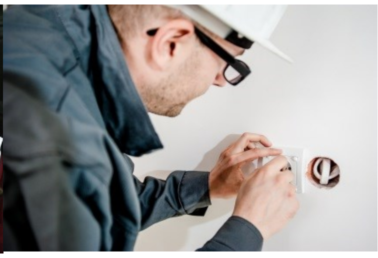
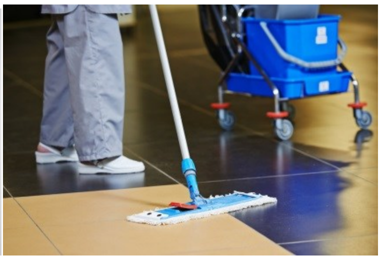

Elective Surgery Centre
Silkeborg Regional Hospital
Silkeborg, Denmark

Danish Ramazzini Centre
Department of Occupational Medicine
Aarhus University Hospital
Aarhus, Denmark

Danish Ramazzini Centre,
Department of Occupational Medicine
Regional Hospital West Jutland –
University Research Clinic
Herning, Denmark

Guidance for home-based shoulder exercises

Exercise movements and difficulty level

- While participating in the research project you are encouraged to exercise 3–4 times a week. During one exercise session, you may perform exercise no. 1 in the morning, exercise no. 2 in the middle of the day, and exercises nos. 3 and 4 in the evening. It is important that you perform all exercises 3–4 times a week.
- The body position from exercise no. 1 must be maintained while performing exercises nos. 2, 3, and 4.
- Exercises nos. 2, 3, and 4 are divided into 3 **difficulty levels: light, medium, and high.** Start with "light" difficulty and increase the difficulty level as range of motion and strength develop.
- When you are able to perform more than 3 sets of 15 repetitions without aggravating your shoulder pain, increase the strength of the elastic band or progress to the next difficulty level. The elastic band colors yellow (light), red (medium) and, green (hard) indicate the strength of the elastic band. Your difficulty level and elastic band color may vary from exercise to exercise.
- All exercises must be performed at a slow and controlled pace, and should be performed for both shoulders.

Pain and tenderness during and after exercise

- During exercise you may experience pain in your shoulder. If the exercise aggravates your shoulder pain and the aggravation does not decrease within 1 hour after exercise, you should decrease one difficulty level. If the exercise still aggravates your shoulder pain and the aggravation does not decrease within 1 hour after exercise, you should also reduce the number of repetitions. Muscle tenderness after exercise is normal and acceptable.

Registration of home-based exercises

- In order to follow your exercises closely, we ask you to complete an exercise diary. Your exercises must also be registered with a BandCizer© sensor, which should be placed on your elastic band during all your exercise sessions (read the BandCizer© instructions at the back of this booklet).

Exercise 1: Posture

This exercise will increase your awareness of posture and give your shoulders a greater range of motion.

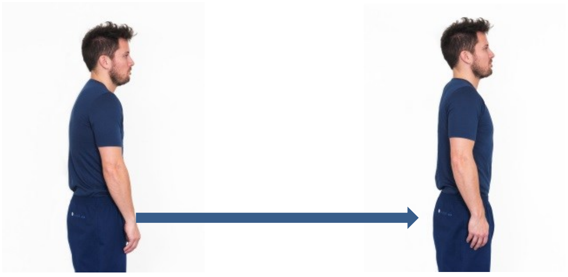

Start position: Stand with a hip width between the feet. Try to "collapse in the back".
End position: Now straighten up and lift up the breastbone. Imagine that you have a cord attached to the top of your head which pulls you up against the ceiling.
Imagine that you will stay long and straight in your back and neck while you retract your shoulders a little. This position should be maintained while doing the following shoulder exercises.

Repeat the exercise 3 times per exercise session. Practice this daily in order to get accustomed to the posture.

Exercise 2: Wall slide – for scapular muscles

Light difficulty:

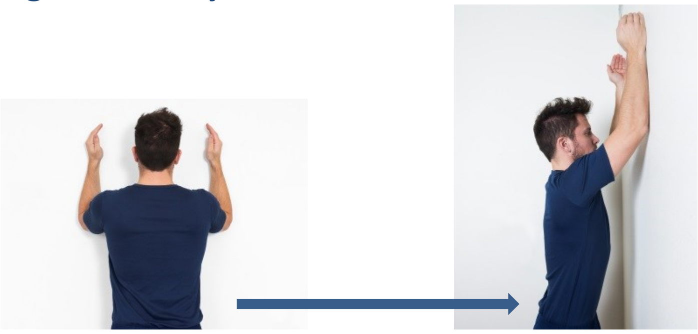

Place the little finger side of your hand and forearm towards the wall (with the shoulders at an angle just below 90°), slide your arms slowly up the wall until the elbows are stretched, thereafter slide down again. The shoulders may lift up slightly towards the ears during the exercise.

Medium difficulty:

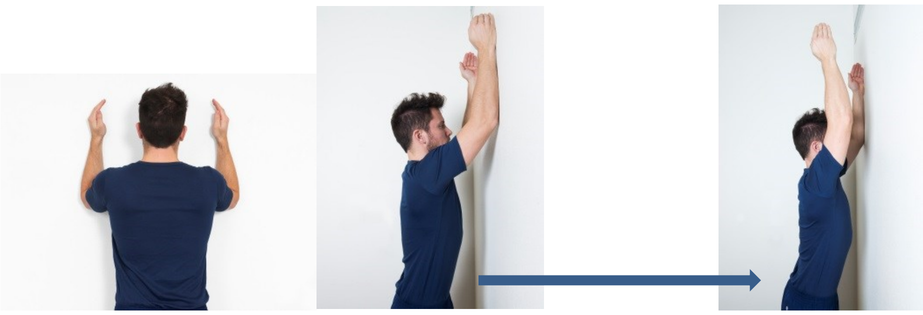

As "Easy difficulty" with addition: when the arms are stretched, lift your right arm a little backwards and place it on the wall again. Thereafter lift your left arm a little backwards and place it on the wall again. Finally, slide down both arms on the wall and start over.

High difficulty:

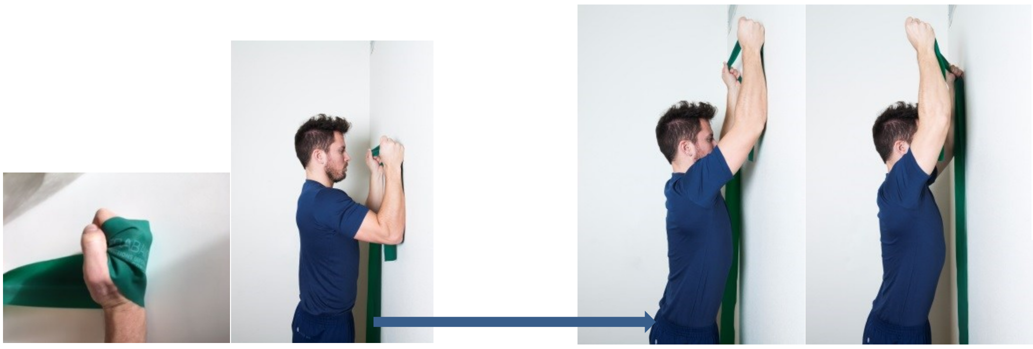

Elastic color:

Yellow
Red
Green

The exercise is performed as "medium difficulty", but with the use of the elastic band. Hold the elastic band in both hands and make sure it is wrapped once around on the hand (see the image of a hand with the elastic band wrapped around). Keep in mind that the little finger side must be held against the wall during the entire exercise.

Exercise 3: Low row / high row – for scapular muscles

Light difficulty:

Elastic color

Yellow
Red
Green

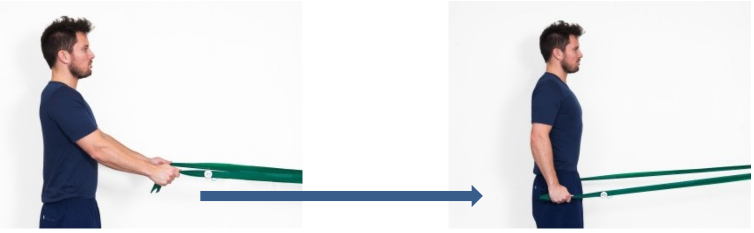

Place the elastic band around a door handle and close the door. Grasp the elastic band with both hands. Move backwards until the elastic band is tightened and the arms are stretched in front of you (the shoulders are at a 45° angle). The elbows are kept stretched throughout the exercise. Now pull the elastic band back slowly until your arms are aligned with your body. Hold the position for a few seconds. Then return to the starting position. Remember the good posture (described in exercise 1).

Medium difficulty:

Elastic color

Yellow
Red
Green

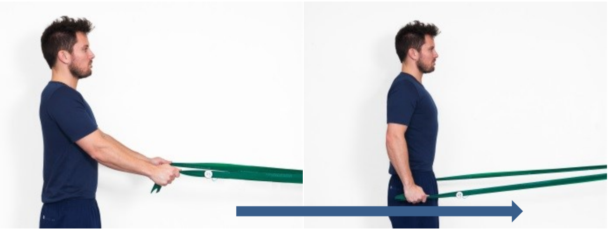

As "Light difficulty", but with an increased strength of the elastic band.

High difficulty:

Elastic color

Yellow
Red
Green

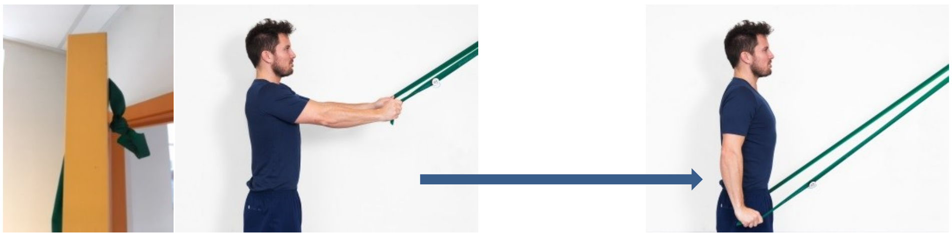

Tie a knot in the middle of the elastic band and attach it to a door (see picture of door with elastic band). Close the door. Grasp the elastic band with both hands. Move away from the door until the elastic band is tightened and your arms are raised to about 90°. The elbows are kept stretched throughout the exercise. Now pull the elastic band back until your arms are aligned with your body. Hold the position for a few seconds. Then return to the starting position.

Exercise 4: External rotation – for rotator cuff muscles

Light difficulty:

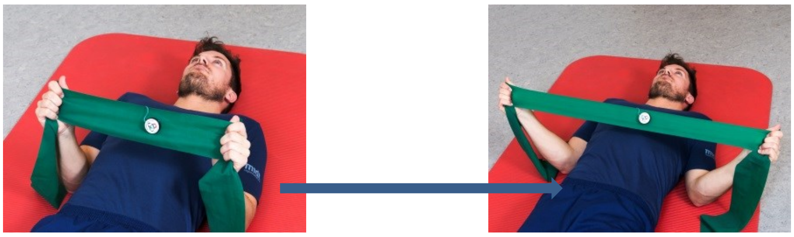

Elastic color

Yellow
Red
Green

Lie on your back with the elastic band around your hands, hold your elbows flexed at 90°, and hold your elbows toward the body during the exercise. Turn the forearms a little outward so the elastic band is strained. Hold the tension for a couple of seconds, then turn the forearms back inwards, so the tension on the elastic band decreases.

Medium difficulty:

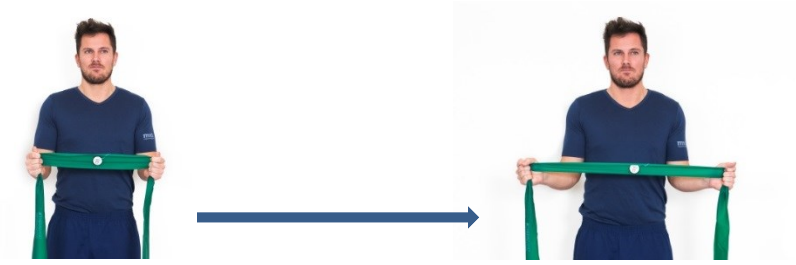

Elastic color

Yellow
Red
Green

Standing up. Place the elastic band around your hands and turn the forearm outwards until the elastic band is strained. Hold the tension for a couple of seconds, then turn the forearms back inwards until the tension of the elastic band decreases.

High difficulty:

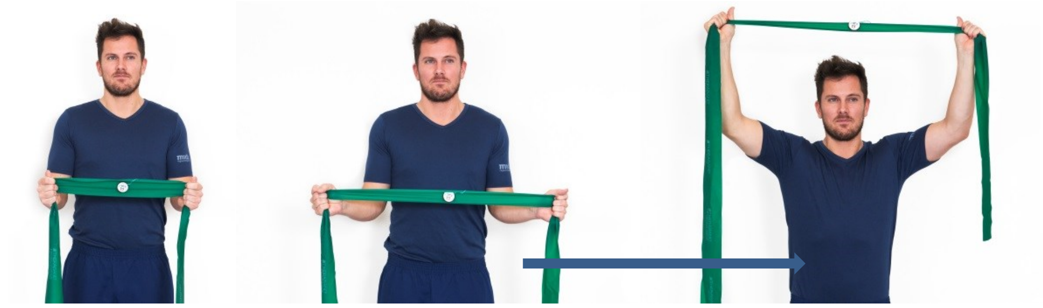

Elastic color

Yellow
Red
Green

Standing up. Place the elastic band around your hands and turn the forearms outwards until the elastic band is strained. Keep the tension on the elastic band and move your arms up over the head and afterwards back down. Then turn the forearms back inwards until the tension on the elastic band decreases.

BandCizer© instruction

In general
The BandCizer© must be put on your elastic band every time you exercise.

Attaching
The BandCizer© is clipped onto the elastic band by its two magnets. During exercise it must be placed in the middle of the elastic band.

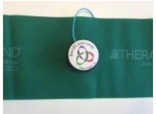

Light and flash
Red flashing means that the BandCizer© detects movement and exercise.
Yellow or violet flashing means that the BandCizer© is in sleep mode and does not detect activity.
When charging, the BandCizer© lights up red, but changes to green when the battery is ready (more than 80% charged).
Fast red or purple flashing indicates low battery level.

If you need to transport the BandCizer©, you must hang the BandCizer© around the elastic band so that it does not register the movement.

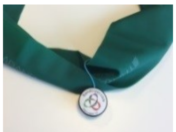

The BandCizer© must be charged regularly – preferably once a week.

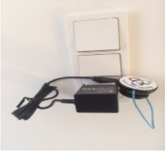

In case of problems with the BandCizer©, please contact
Jeanette Trøstrup (phone number: 24 75 91 53; email: jeatro@rm.dk)

Elective Surgery Centre
Silkeborg Regional Hospital
Silkeborg, Denmark

Danish Ramazzini Centre
Department of Occupational Medicine
Aarhus University Hospital
Aarhus, Denmark

Danish Ramazzini Centre,
Department of Occupational Medicine
Regional Hospital West Jutland –
University Research Clinic
Herning, Denmark
