# Supplementary material for: Reducing shoulder complaints in employees with high occupational shoulder exposures: study protocol for a cluster-randomised controlled study (The Shoulder-Café Study)
Source: Trials. 2019 Nov 12;20:627. doi: 10.1186/s13063-019-3703-y (PMC6852773; doi:10.1186/s13063-019-3703-y)
Supplement: Supplementary file 3 — Additional file 3. Pamphlet – How to reduce occupational shoulder exposures. (Images are our own). [file 13063_2019_3703_MOESM3_ESM.pdf]

Additional file 3

The law says

All work tasks must be planned, organised, and performed in a safe manner.

Contact

Jeanette Trøstrup
Elective Surgery Centre
Silkeborg Regional Hospital
Denmark

How to reduce occupational shoulder exposures

In this pamphlet you can read about the different types of occupational shoulder exposures and how to reduce them.

Work with high shoulder exposures include

- Work with elevated arms
- Work with repetitive shoulder movements
- Work with forceful shoulder exertions

Work with elevated arms
Work where one or both elbows are lifted to around or above shoulder height (90°).

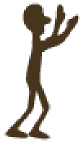

Work with elevated arms often occurs in e.g.

- House painters
- Electricians
- Insulation workers
- Scaffolders
- Carpenters

How is the exposure reduced?

- Reduce the amount of time spent with overhead tasks.
- Use technical aids wherever possible.
- Design the workplace appropriately and ensure adequate work space.

Work with repetitive shoulder movements
Work with fast repetitive shoulder movements, which is performed for a large part of the working day.

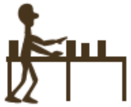

Work with repetitive shoulder movements often occurs in e.g.

- Manual packers
- Laundry workers
- Cleaners
- Kitchen assistants
- Bakers

How is the exposure reduced?

- Use technical solutions, e.g. machines or robots to perform repetitive tasks.
- Reduce time with repetitive work, e.g. by rotation, so that the repetitive work is distributed among several workers.

Work with forceful shoulder exertions
Work which is physically demanding for the shoulders, e.g. lifting, pulling, and pushing.

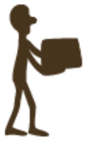

Work with forceful shoulder exertions often occurs in e.g.

- Scaffolders
- Slaughterhouse workers
- Wood industry workers
- Bricklayers’ assistants
- Poultry processing workers
- Carpenters

How is the exposure reduced?

- Plan and organise the work, e.g. order and use items that weigh less.
- Use technical solutions, e.g. lift, crane, hoist, and similar equipment for handling heavy items.
- Heavy items should be lifted by two or more workers.
