# Supplementary material for: Reducing shoulder complaints in employees with high occupational shoulder exposures: study protocol for a cluster-randomised controlled study (The Shoulder-Café Study)
Source: Trials. 2019 Nov 12;20:627. doi: 10.1186/s13063-019-3703-y (PMC6852773; doi:10.1186/s13063-019-3703-y)

Additional file 4: Individual feedback on occupational shoulder exposures

First feedback-letter:

Dear xxxx,

After our first meeting you have been working 1–5 days with the Axivity attached to your shoulder and you have filled out a work diary.

Based on your measurements and diary, you are medium exposed to work with elevated arms (> 60° and > 90°) and repetitive shoulder movements and low exposed to forceful shoulder exertions. If you have any questions, feel free to contact us.

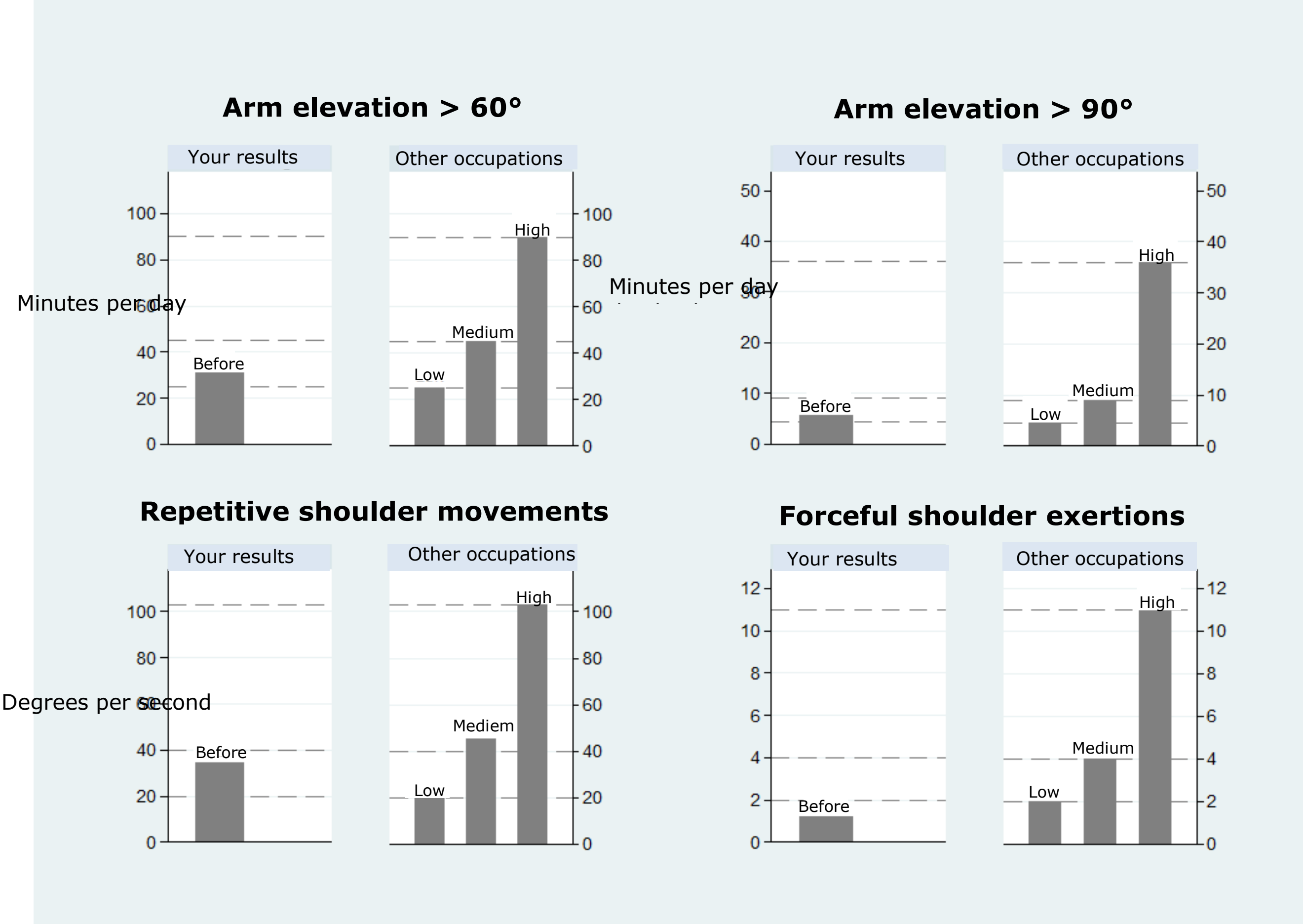

Additional file 4: Individual feedback on occupational shoulder exposures

Second feedback-letter:

Dear xxxx,

Thank you for participating in the scientific project "Reducing shoulder complaints and shoulder exposures".

We have analysed your second Axivity measurements and work diary. The figures below illustrate the results of your measurements. You can compare your second measurements with your first measurements (before and after).

You are welcome to contact Jeanette Trøstrup if you have any questions
(phone number 2475 9153; or email: jeatro@rm.dk).

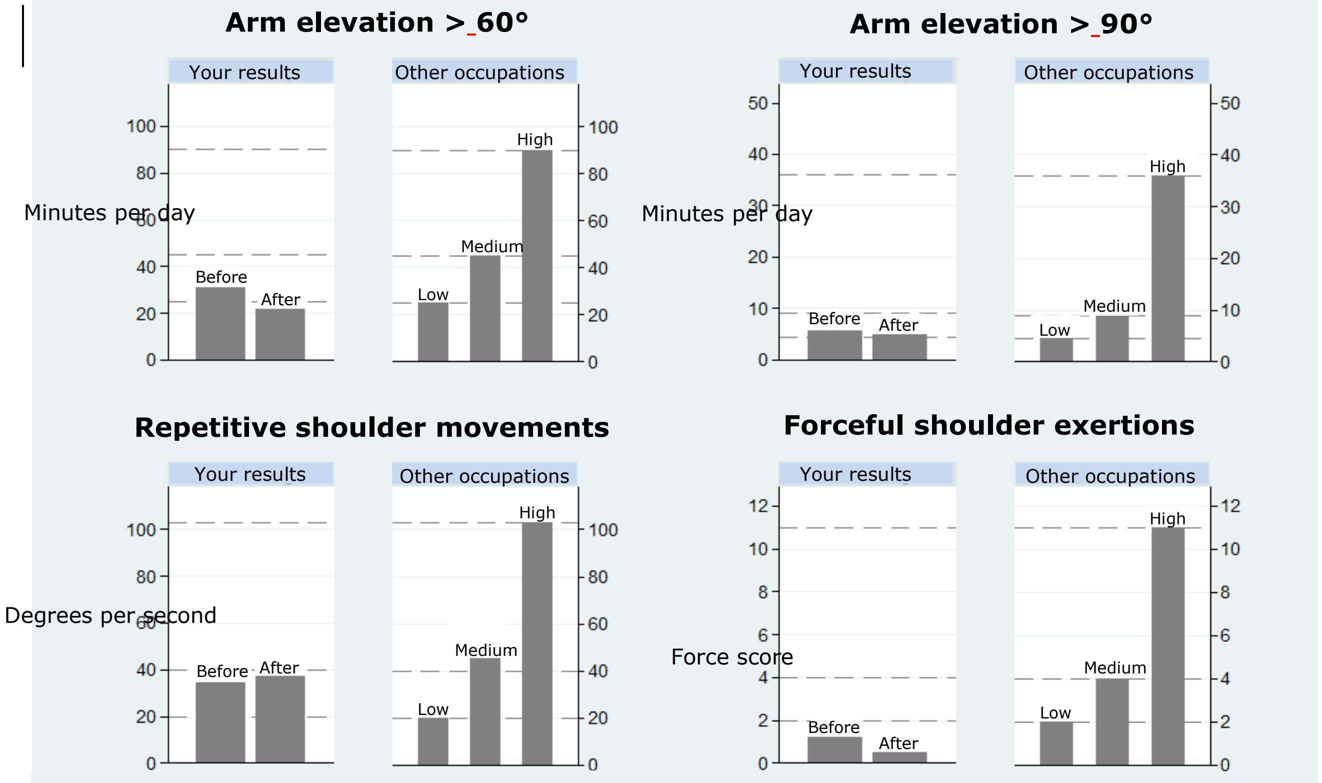

Supplement: Supplementary file 4 — Additional file 4. Individual feedback on occupational shoulder exposures. [file 13063_2019_3703_MOESM4_ESM.pdf]
