# Supplementary material for: Reducing shoulder complaints in employees with high occupational shoulder exposures: study protocol for a cluster-randomised controlled study (The Shoulder-Café Study)
Source: Trials. 2019 Nov 12;20:627. doi: 10.1186/s13063-019-3703-y (PMC6852773; doi:10.1186/s13063-019-3703-y)
Supplement: Supplementary file 5 — Additional file 5. Guideline for supervised exercises. [file 13063_2019_3703_MOESM5_ESM.pdf]

Guideline for supervised exercises

At each café meeting, approximately one hour is allocated for exercise supervision carried out by the municipal physiotherapists.

How to supervise:

- The municipal physiotherapist demonstrates each exercise in plenum to all participants and each participant gets the opportunity to perform the exercise a few times supervised by the physiotherapist.
- Participants are advised to perform the exercises according to the instructions and recommendations below.
- The participant is instructed in use of the BandCizer© sensor.
- The participant is instructed in how to fill out the exercise diary correctly.

How the exercises should be performed:

- The dynamic exercises (exercises 2-4) must be performed at a slow and controlled pace: The exercises should be performed with two seconds in both concentric and eccentric phase.
- All participants must start with "Light difficulty".
- When the participant can perform 3 sets of 15 repetitions with good control and without provocation of shoulder pain, the participant should progress to the next exercise level. First, increase the elastic band colour from yellow, through red to green and then go to the next level of the exercise.
- During exercise, pain may be expected in the shoulder. If the aggravation does not decrease to the level before exercise (within max 1 hour), the participant should decrease to a lower elastic band colour or lower the difficulty level. If the exercise still aggravates pain, the number of repetitions shoulder be reduced. Muscle tenderness after exercise is normal and acceptable.
- All exercises must be performed for both shoulders.

Recommendations given to the participants about the training:

- Read the pamphlet "Home-based shoulder exercise programme".
- The exercises should be performed at home 3−4 times weekly throughout the intervention period (around 3 months). It is further recommended, that the exercises are performed at least until 3 months after end of intervention.
- The BandCizer© should only be used during the intervention period (afterwards it must be returned to Jeanette Trøstrup).
- The participant is encouraged to perform the exercises in front of a mirror.
- The duration of the home-based exercises is expected to be approximately 15 minutes per session.
