# Supplementary material for: Reducing shoulder complaints in employees with high occupational shoulder exposures: study protocol for a cluster-randomised controlled study (The Shoulder-Café Study)
Source: Trials. 2019 Nov 12;20:627. doi: 10.1186/s13063-019-3703-y (PMC6852773; doi:10.1186/s13063-019-3703-y)
Supplement: Supplementary file 6 — Additional file 6. Clinical shoulder examination form. [file 13063_2019_3703_MOESM6_ESM.pdf]

Additional file 6: Clinical shoulder evaluation form

Participant (name) ________________________________ Participant ID___ ___ ___ ___ ___ ___ ___ ___

Date: __ __ / __ __ - __ __ __ __ Physiotherapist initials: ___ ___ ___

History

For how long have you experienced pain? (week, month, year) ____

Is the pain caused by trauma: □ No
□ Yes

Other symptoms: □ No
□ Yes, which: weight loss / difficulty breathing / coughing / expectoration of sputum / fever / dizziness / headache / fatigue

Additional history (if necessary)

Current symptoms: neck / shoulder blade / shoulder / upper arm / elbow / forearm / hand R/L

Pain: constant / intermittent / nightly ____

What aggravates / improves: ____

Pain medication: ____

Other treatment: ____

Work / sport / leisure: ____

Other diseases: ____

Other:____

Clinical findings

**Inspection**	**Right**		**Left**	
Atrophy	□ No<br>□ Yes, located	□ Infraspinatus<br>□ Supraspinatus<br>□ Deltoid<br>□ Other:______________	□ No<br>□ Yes, located:	□ Infraspinatus<br>□ Supraspinatus<br>□ Deltoid<br>□ Other:________________
Shoulder malalignment	□ No<br>□ Yes, malalignment:______________________		□ No<br>□ Yes, malalignment:______________________	
**Col. cerv.**	**Right**		**Left**	
Range of motion	□ Normal	□ Decreased<br>□ Pain<br>Describe:_____________	□ Normal	□ Decreased<br>□ pain<br>Describe:________________
Foramen compression test	□ Negative	□ Positive	□ Negative	□ Positive
**Shoulder**	**Right**		**Left**	
Scapulohumeral rhythm	□ No scapular dyskinesia<br>□ Scapular dyskinesia, describe:______________		□ No scapular dyskinesia<br>□ Scapular dyskinesia, describe:_________________	
Painful arc test	□ Negative<br>□ Positive: □ 60 - 120° and/or □ > 120°		□ Negative<br>□ Positive: □ 60 - 120° and/or □ > 120°	
AROM/PROM	□ AROM	□ PROM	□ AROM	□ PROM
Flex:	____________°	____________°	____________°	____________°
Abd:	____________°	____________°	____________°	____________°
Ext rot:	____________°	____________°	____________°	____________°
Int rot:	□ Shoulder blade<br>□ TH 12<br>□ Waist<br>□ SI-joint<br>□ Buttock<br>□ Thigh	____________°<br>____________°<br>____________°<br>____________°<br>____________°<br>____________°	□ Shoulder blade<br>□ TH 12<br>□ Waist<br>□ SI-joint<br>□ Buttock<br>□ Thigh	____________°<br>____________°<br>____________°<br>____________°<br>____________°<br>____________°
**Palpation**	**Right**		**Left**	
Tenderness	□ No<br>□ Yes, located:	□ Sulcus<br>□ Sternoclav-joint<br>□ AC-joint<br>□ GH-joint<br>□ Muscular: Trapez / Lev. sca / IS / SS / Pec.major / Regio nuc.	□ No<br>□ Yes, located:	□ Sulcus<br>□ Sternoclav-joint<br>□ AC-joint<br>□ GH-joint<br>□ Muscular: Trapez / Lev. sca / IS / SS / Pec.major / Regio nuc.

Reduced strength	Right			Left		
Elev / flex	□ No	□ Yes		□ No	□ Yes	
Abd / add	□ No	□ Yes		□ No	□ Yes	
Ext rot	□ No	□ Yes		□ No	□ Yes	
Int rot	□ No	□ Yes		□ No	□ Yes	
**Pain when moving against resistance**	**Right**			**Left**		
Flex / ext	□ No	□ Yes		□ No	□ Yes	
Abd / add	□ No	□ Yes		□ No	□ Yes	
Ext rot	□ No	□ Yes		□ No	□ Yes	
Int rot	□ No	□ Yes		□ No	□ Yes	
**Specific tests**	**Right**			**Left**		
Jobe's	□ Negative	□ Positive		□ Negative	□ Positive	
Hawkins'	□ Negative	□ Positive		□ Negative	□ Positive	
Hawkins' modified	□ Negative	□ Positive		□ Negative	□ Positive	
Neer's	□ Negative	□ Positive		□ Negative	□ Positive	
O'Brien	□ Negative	□ Positive		□ Negative	□ Positive	
Cross over	□ Negative	□ Positive		□ Negative	□ Positive	
Scapula Assistance test	□ Negative	□ Positive		□ Negative	□ Positive	
Scapula Retraction Test	□ Negative	□ Positive		□ Negative	□ Positive	
**Additional tests (performed if necessary)**						
Yergason	□ Negative	□ Positive		□ Negative	□ Positive	
Apprehension	□ Negative	□ Positive	___________°	□ Negative	□ Positive	___________°
Relocation	□ Negative	□ Positive		□ Negative	□ Positive	
Sulcus	□ Negative	□ Positive		□ Negative	□ Positive	
Belly pres	□ Negative	□ Positive		□ Negative	□ Positive	
Hornblower's	□ Negative	□ Positive		□ Negative	□ Positive	
**Comments to tests**						

Categorisation

□ Subacromial impingement syndrome
□ Muscle tenderness
□ Frozen shoulder
□ Rotator-cuff-rupture
□ Suspicion about nerve injury
□ AC-joint problems
Instability, conditional on:
□ Hypermobility
□ Trauma
□ Other: ____

Treatment plan

□ Exercise
□ No exercise
□ Advised to contact general practitioner

Comments

____
____
____

Abbreviations:
Abd = Abduction
AC = Acromioclavicular
Add = Adduction
AROM = Active range of motion
Elev = Elevation
Ext = External
Flex = Flexion

GH = Glenohumeral
Int = Internal
IS = Infraspinatus
Lev.sca = Levator scapulae
Pec.major = Pectoralis major
Regio nuc = Regio nuchae
PROM = Passive range of motion
SI = Sacroiliac
SS = Supraspinatus
Sternoclav = sternoclavicular
TH 12 = Twelfth thoracic vertebra

* A clinical shoulder evaluation manual can be requested in Danish from Jeanette Trøstrup (jeatro@rm.dk).
