# Supplementary material for: Reducing shoulder complaints in employees with high occupational shoulder exposures: study protocol for a cluster-randomised controlled study (The Shoulder-Café Study)
Source: Trials. 2019 Nov 12;20:627. doi: 10.1186/s13063-019-3703-y (PMC6852773; doi:10.1186/s13063-019-3703-y)
Supplement: Supplementary file 7 — Additional file 7. Educational slides – shoulder anatomy. (Images on page 3 were bought from Colorbox. Other photos are our own). [file 13063_2019_3703_MOESM7_ESM.pdf]

Additional file 7: Educational slide – Shoulder. (Images side 3 is bought from Colorbox)

Education program

Causes of shoulder complaints

Anatomy

Pain and exercises

Clinical shoulder evaluation

Causes of shoulder complaints

Age, genetics, lifestyle, workloads

Different tissues can be painful: joints, muscles, tendons, bursa

Muscular stability and posture of the shoulder, shoulder blade, and upper back

Shoulder anatomy

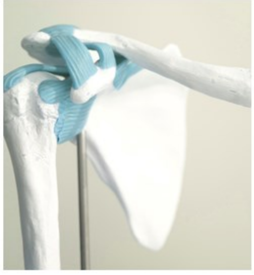

Posture

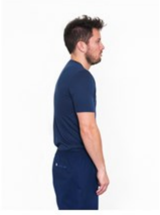

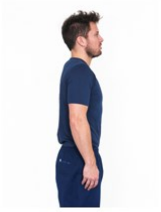

Pain and exercise

Known pain must not be aggravated for more than 1 hour after exercise

Muscle tenderness is natural and expected

Clinical shoulder evaluation

Purpose:

Evaluate shoulder problem

Make sure that it is advisable for participant to exercise

Give short individual guidance

Guidance for home exercise

Exercise 3-4 times a week

The exercise may be divided into several periods a day

Keep the posture (exercise 1) in exercises 2, 3, and 4

Exercises level and dose

- Exercises 2, 3, and 4 have three levels
- Start with level one and perform as many repetitions as possible
- If you can perform > 3x15 repetitions with good control and without aggravation of shoulder pain → increase to next level
- If your shoulder pain is aggravated and if the pain does not return to the starting level (within 1 hour) → decrease one level
