# Supplementary material for: Reducing shoulder complaints in employees with high occupational shoulder exposures: study protocol for a cluster-randomised controlled study (The Shoulder-Café Study)
Source: Trials. 2019 Nov 12;20:627. doi: 10.1186/s13063-019-3703-y (PMC6852773; doi:10.1186/s13063-019-3703-y)

Additional file 8: Educational slide – Workplace councelling

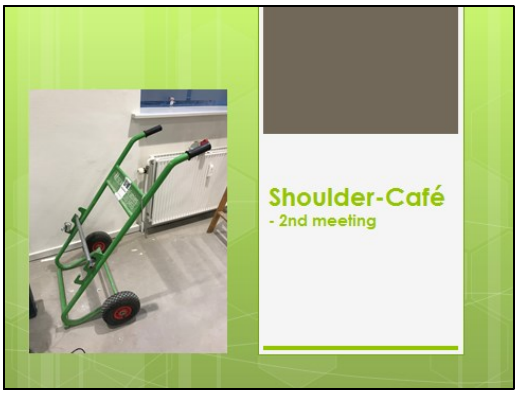

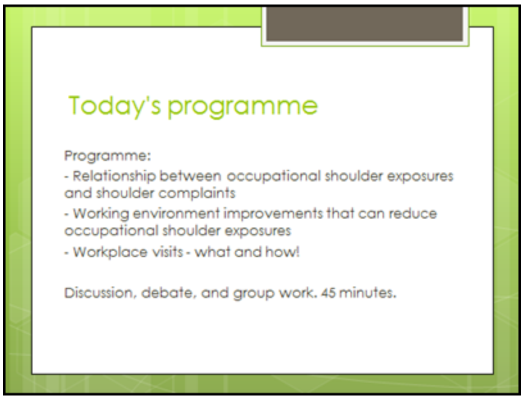

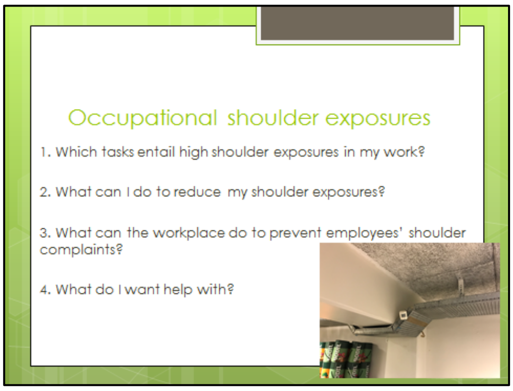

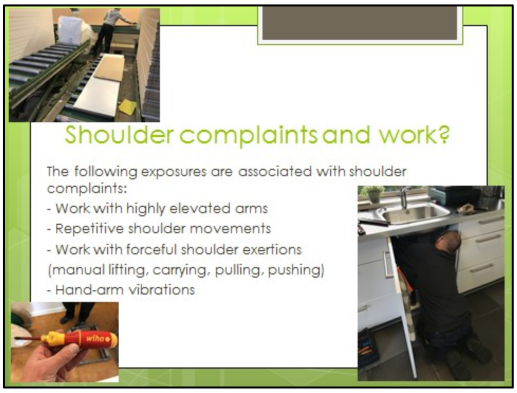

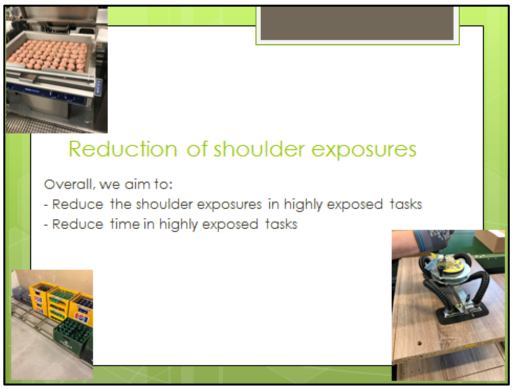

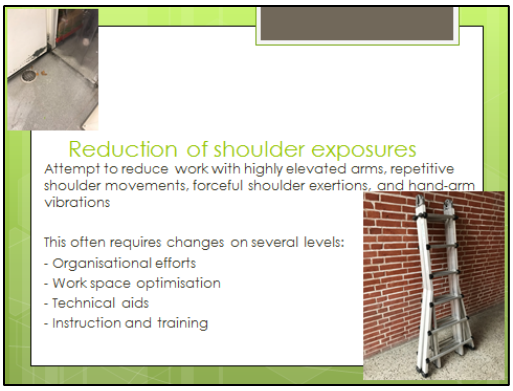

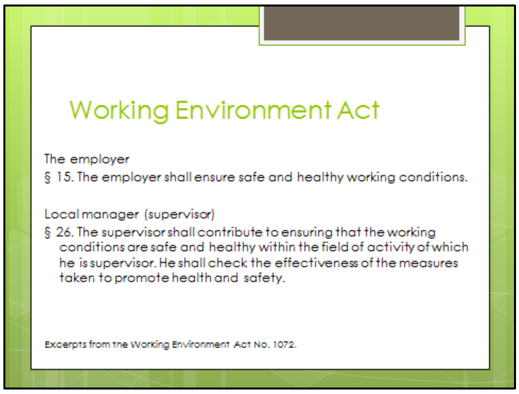

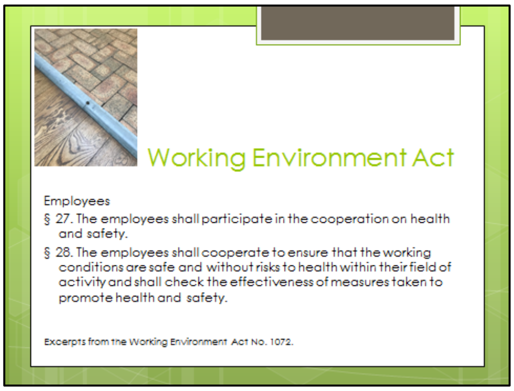

Additional file 8: Educational slide – Workplace councelling

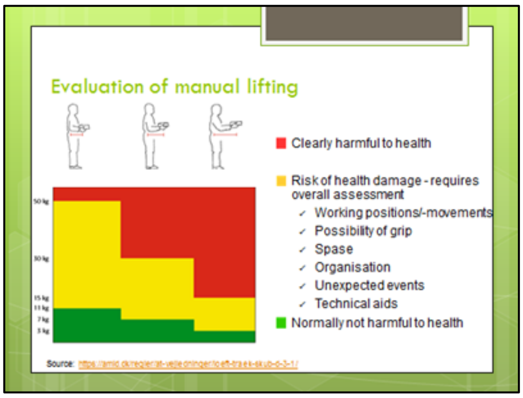

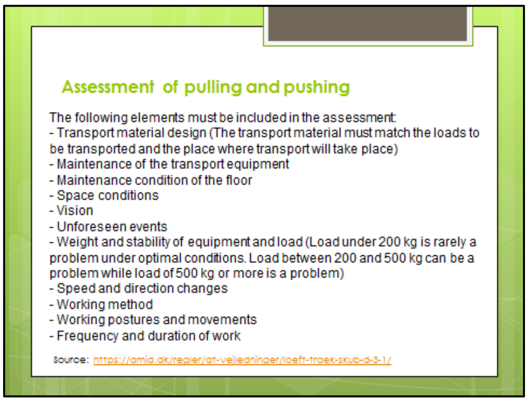

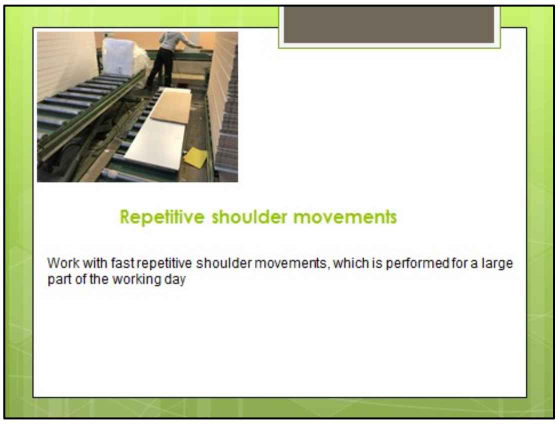

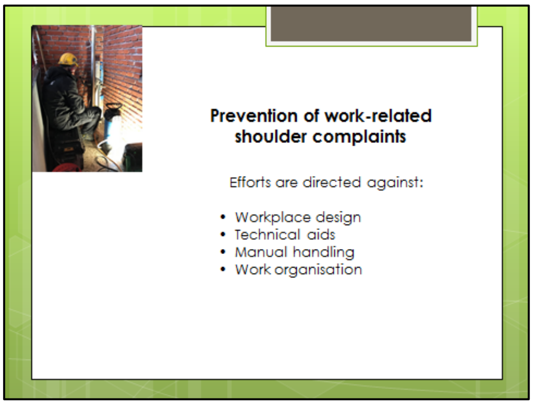

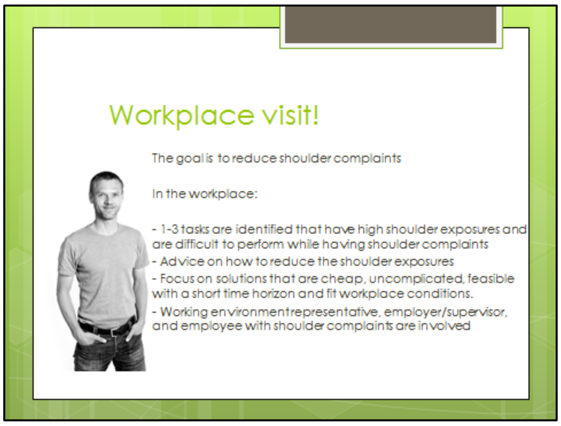

Supplement: Supplementary file 8 — Additional file 8. Educational slides – workplace counselling. (Images are our own). [file 13063_2019_3703_MOESM8_ESM.pdf]
