# Supplementary material for: Reducing shoulder complaints in employees with high occupational shoulder exposures: study protocol for a cluster-randomised controlled study (The Shoulder-Café Study)
Source: Trials. 2019 Nov 12;20:627. doi: 10.1186/s13063-019-3703-y (PMC6852773; doi:10.1186/s13063-019-3703-y)
Supplement: Supplementary file 9 — Additional file 9. Statistical analysis plan (SAP). [file 13063_2019_3703_MOESM9_ESM.pdf]

Statistical analysis plan (SAP)

Reducing shoulder complaints in employees with high occupational shoulder exposures: a cluster-randomised controlled study (The Shoulder-Café Study)

ClinicalTrials.gov ID: NCT03159910

Protocol version 1.0, SAP version 1.0, 10 January 2019

Names, affiliations and roles of SAP contributors

Jeanette Trøstrup, Elective Surgery Centre, Silkeborg Regional Hospital, Silkeborg, Denmark; principal investigator

Date and signature: 1/10/2019 Jeanette Trøstrup

Morten Frydenberg, Danish Ramazzini Centre, Department of Occupational Medicine, Regional Hospital West Jutland – University Research Clinic, Herning, Denmark; statistical advisor

Date and signature: 1/10/2019 [illegible]

Poul Frost, Danish Ramazzini Centre, Department of Occupational Medicine, Aarhus University Hospital, Aarhus, Denmark; investigator

Date and signature: 1/10/2019 [illegible]

Susanne Wulff Svendsen, Danish Ramazzini Centre, Department of Occupational Medicine, Regional Hospital West Jutland – University Research Clinic, Herning, Denmark; chief investigator

Date and signature: 1/10/2019 Susanne Wulff Svendsen

INTRODUCTION

Please refer to the study protocol regarding background and rationale. This trial compares a group-based Shoulder-Café intervention with an individual-based Shoulder-Guidance intervention (active control – enhanced usual care). The main hypotheses are that the Shoulder-Café will reduce I) shoulder complaints and II) occupational shoulder exposures more effectively than the Shoulder-Guidance. The trial results for hypotheses I and II will be reported in two separate papers.

METHODS

Design

The trial uses a cluster-randomised controlled design with two parallel interventions: Shoulder-Café and Shoulder-Guidance. The intervention duration is around 3 months.

Population and randomisation

Details on screening, eligibility, and recruitment including a template flow diagram are provided in the study protocol. According to the sample size calculation in the study protocol, the study size needs to be ≥96 (2 x 48). We aim to include 60 employees in each group to ensure that 50 employees in each group complete.

Randomisation is performed at company (cluster) level with a 1:1 allocation ratio and stratified by industry using blocking within strata with randomly permuted block sizes of 2-4-6. Blocking within strata is used to ensure an equal distribution of the interventions between industries, while randomly permuted block sizes ensure allocation concealment. The randomisation result is not revealed to the participants, until they have signed the informed consent and completed the baseline questionnaire.

Baseline assessment of occupational shoulder exposures is performed after the randomisation result has been revealed.

The analysis population for hypotheses I and II consists of all participants with baseline questionnaire information (including a valid Oxford Shoulder Score (OSS)) and randomised allocations, who have not withdrawn their consent to contribute to the study at any time between inclusion and the first submission of a manuscript to a scientific journal. All analyses will be based on the analysis population according to the intention-to-treat principle.

Study outcomes

Details on primary, secondary, and supplementary outcomes are provided in the study protocol. Regarding hypothesis I, the primary outcome is the OSS at 6 months. Listed in order of priority, the secondary outcomes are: the OSS at 12 months, the Fear Avoidance Beliefs Questionnaire – Physical Activity (FABQ-PA) at 6 months, the Patients' global impression of change (PGIC) at 6 months, and the FABQ-PA at 12 months.

Regarding hypothesis II, the primary outcome is the mean number of minutes/day working with the arm elevated > 60˚ shortly after end of intervention (EOI). Measurement days, which fulfill the quality requirement of ≥ 4 hours/day, are normalised to the participant's scheduled working hours that day according to the work diary, and the outcome is calculated as the total number of minutes working with the arm elevated > 60˚ across the measurement days for each participant divided by the number of measurement days for the participant (1–5 days). the number of measurement days for the participant (1–5 days). Listed in order of priority, the secondary outcomes are: the mean number of minutes/day working with the arm elevated > 90˚ shortly after EOI, calculated as

described above, the mean median angular velocity (˚/s) shortly after EOI, calculated as the mean of the medians for each measured working day for each participant, the Borg CR-10 shortly after EOI, calculated as the mean of the rated working days for each participant, and the mean number of minutes/day working with the arm elevated > 30˚ shortly after EOI, calculated as described for the primary outcome.

Schedule for study procedures

The schedule for study procedures including assessment of primary, secondary, and supplementary outcomes is presented in the study protocol. Regarding hypothesis I, follow-up takes place 6 and 12 months after T_0 = start of intervention; regarding hypothesis II, follow-up takes place shortly after end of intervention (EOI). The interventions are based on non-invasive methods and are not expected to cause any adverse events other than possible temporary muscle tenderness after shoulder exercises. Therefore, no interim analyses are planned and no stopping rules defined.

Characteristics of non-participants

Employees may decline to participate any further at two steps of the recruitment process; step 1) the screening questionnaire and step 2) the telephone interview (see the template flow diagram in the study protocol). At each of these two steps, we will compare those who agreed to participate with those who declined based on data from the screening questionnaire (age, sex, industry, and the OSS). Regarding hypothesis II, participants and non-participants will also be compared with respect to self-reported occupational shoulder exposures at baseline.

Baseline characteristics of the analysis population

Continuous variables will be summarised by mean and standard deviation (or median and inter quartile range if data is skewed); categorical variables will be summarised by number and percentage. Baseline characteristics of the analysis population will be summarised and presented as illustrated in Tables 1A (hypothesis I) and 1B (hypothesis II).

Adherence

For the analysis population and the population completing follow up, adherence to the home-based exercise programme will be described according to intervention arm as the mean (SD) number of days/person where exercises were performed during the intervention period according to the exercise diary and BandCizer© recordings. Adherence to the exposure assessment will be described according to intervention arm as the percentage of the analysis population that has ≥ 1 work day with ≥ 4 hours of Axivity data and/or a Borg-CR10 rating 1) shortly after T_0 and 2) shortly after EOI. For the Shoulder-Café group, adherence to café-meetings will be described as the percentages of the participants who completed three, two, or only one café meeting. For the Shoulder-Guidance group, adherence to intervention contacts will not be further described because all participants have to attend the 1st individual appointment to be included and are not scheduled to further contacts.

Table 1A Baseline characteristics of the analysis population regarding hypothesis I.

**Population**	**Analysis population**		**Population with follow-up at 6 months**		**Population with follow-up at 12 months**	
	**Shoulder-Café n**	**Shoulder-Guidance n**	**Shoulder-Café n**	**Shoulder-Guidance n**	**Shoulder-Café n**	**Shoulder-Guidance n**
Age, mean (SD)						
Sex, n (%)						
Female						
Male						
Industry, n (%)						
Service						
Manufacture						
Construction						
OSS, mean (SD)						
FABQ-PA, mean (SD)						

Abbreviations: OSS = Oxford Shoulder Score, FABQ-PA = Fear Avoidance Beliefs Questionnaire – Physical Activity.

Table 1B Baseline characteristics of the participants regarding hypothesis II.

Population	Participants with exposure assessment shortly after T_0		Participants with exposure assessment shortly after T_0 and shortly after EOI	
	**Shoulder-Café n**	**Shoulder-Guidance n**	**Shoulder-Café n**	**Shoulder-Guidance n**
Age, mean (SD)				
Sex, n (%)				
Female				
Male				
Industry, n (%)				
Service				
Manufacture				
Construction				
OSS, mean (SD)				
Arm elevation (minutes/day), mean (SD)				
> 60°				
> 90°				
Repetitive shoulder movements (median angular velocity, °/s), mean (SD)				
Forceful shoulder exertions (Borg CR-10), mean (SD)				
Arm elevation (minutes/day), mean (SD) > 30°				

Abbreviation: EOI = end of intervention, OSS = Oxford Shoulder Score.

Statistical principles and analyses

Regarding hypothesis I, a mixed model analysis of the OSS will be performed including "intervention" (Shoulder-Café and Shoulder-Guidance), "time" (6 and 12 month follow-up), "intervention x time", baseline OSS (linear), sex, age (linear), and industry (service, manufacture, construction) as fixed effects, adjusting for random effects of participant and company (cluster); the effect estimate will be the mean difference (Shoulder-Café minus Shoulder-Guidance) at each time point, reported with a 95% confidence interval (CI). FABQ-PA will be analysed likewise, but will be adjusted for baseline FABQ-PA instead of baseline OSS. In the analysis of PGIC, we will dichotomise the outcome as improved (no/yes) and use a risk difference model if around 50% of the participants improve. If a considerably smaller percentage (< 20%) improves, we will employ a relative risk model (log-binomial model) using improved as the outcome, while if a considerably larger percentage (> 80%) improves, we will employ a relative risk model using not improved as the outcome. The analysis of PGIC will be adjusted for sex, age, and industry and use robust standard errors to take into account clustering at company level.

Regarding hypothesis II, a mixed model analysis of the primary outcome (minutes/day working with the arm elevated > 60˚ shortly after EOI) will be performed including "intervention" (Shoulder-Café and Shoulder-Guidance), baseline minutes/day working with the arm elevated > 60˚ (linear), sex, age (linear), and industry (service, manufacture, construction) as fixed effects, adjusting for random effects of company (cluster); the primary effect estimate will be the mean difference (Shoulder-Café minus Shoulder-Guidance), reported with a 95% CI. The analyses for the secondary outcomes will be performed likewise, but will be adjusted for the respective baseline values instead of the baseline number of minutes/day working with the arm elevated > 60˚.

Results for continuous outcomes will be presented as adjusted values, but unadjusted mean values will also be shown, see tables 2A and 2B; results for PGIC will be presented in text. All CIs will be bootstrapped so that they will be robust to deviations from distributional assumptions; this will also minimise effects of outlying outcome measures, which is already minimised because we include baseline measures in the analyses. No adjustment for multiplicity is planned. We do not intend to perform per-protocol and subgroup analyses. Regarding hypothesis II, we will perform sensitivity analysis, where we exclude working days with unusual shoulder exposures according to the work diary (e.g., if a person reports unusual shoulder exposures a given working day, the mean exposure will be based on the remaining working days with usual shoulder exposures).

Usual missing rules for the OSS will be used. Numbers of participants with missing data will be reported. Imputation will not be performed as we have no extra information that is not already included in the mixed model, which will account for missing values that are missing completely at random given the variables included in the model.

If more than 5% of the primary outcome measures in any of the intervention groups are missing, we will undertake sensitivity analyses of the primary outcomes to evaluate any effects of differential loss to follow-up. Regarding hypotheses I and II, missing values of the OSS at 6 and 12 months and missing minutes/day > 60˚ shortly after EOI will be checked using the model based predicted values going through more scenarios: Shoulder-Café + 0 SD and Shoulder-Guidance + 1 SD; Shoulder-Café + 0 SD and Shoulder-Guidance - 1 SD; Shoulder-Café + 1 SD and Shoulder-Guidance + 0 SD, and Shoulder-Café - 1 SD and Shoulder-Guidance + 0 SD.

Axivity data (Axivity Ltd, Newcastle, United Kingdom) will be downloaded using OmGui open-source software (OmGui Version 1.0.0.28; Open Movement, Newcastle University, Newcastle upon Tyne, United Kingdom) and saved in raw format files. MatLab (Build 8.6.0.267246 (R2015b) 64 bit) and STATA 15 (StataCorp LP, College Station, TX, US) will be used for data processing and statistical analyses. Data cleaning will be documented in Stata do files.

The final analyses are planned to take place when 12 month follow-up has been reached for all participants and when the data has been cleaned. The paper regarding hypothesis I is expected to be prepared around August 2020 and the paper regarding hypothesis II shortly thereafter.

We have published our study protocol including this SAP to minimise the risk of analysis bias.

Table 2A Effectiveness Shoulder-Café compared with Shoulder-Guidance with respect to primary and secondary outcomes (hypothesis I).

	**Shoulder-Café**	**Shoulder-Guidance**	**Effectiveness**	
	**n**	**n**	**Mean difference ***	**95% CI**
**Primary outcome**				
OSS at 6 months, mean (SD)				
**Secondary outcomes**				
OSS at 12 months, mean (SD)				
FABQ-PA at 6 months, mean (SD)				
FABQ-PA at 12 months, mean (SD)				

* Adjusted for the baseline value of the relevant outcome, sex, age, and industry using mixed models including company and participant as random effects.

Abbreviations: CI = confidence interval, FABQ-PA = Fear Avoidance Beliefs Questionnaire – Physical Activity, OSS = Oxford Shoulder Score, PGIC = Patients' Global Impression of Change.

Table 2B Effectiveness of Shoulder-Café compared with Shoulder-Guidance with respect to primary and secondary outcomes (hypothesis II).

	**Shoulder-Café**			**Shoulder-Guidance**			**Effectiveness**	
	**n**	**Mean**	**SD**	**n**	**Mean**	**SD**	**Mean difference***	**95% CI**
**Primary outcome**								
Arm elevation (minutes/day) > 60°								
**Secondary outcomes**								
Arm elevation (minutes/day) > 90°								
Repetitive shoulder movements (median angular velocity, °/s)								
Forceful shoulder exertions (Borg CR-10)								
Arm elevation (minutes/day) > 30°								

* Adjusted for the baseline value of the relevant outcome, sex, age (linear), and industry (service, manufacture, construction) as fixed effects, and random effect of company (cluster).

Abbreviation: CI = confidence interval.
