# Supplementary material for: Reducing shoulder complaints in employees with high occupational shoulder exposures: study protocol for a cluster-randomised controlled study (The Shoulder-Café Study)
Source: Trials. 2019 Nov 12;20:627. doi: 10.1186/s13063-019-3703-y (PMC6852773; doi:10.1186/s13063-019-3703-y)
Supplement: Supplementary file 10 — Additional file 10. List of questionnaires and other documents used in the project. [file 13063_2019_3703_MOESM10_ESM.pdf]

Additional file 10: List of questionnaires and other documents used in the project

Questionnaires:

- Screening questionnaire
- Questionnaire A: Baseline
- Questionnaire B: 6 month follow-up
- Questionnaire C: 12 month follow-up
- Questionnaire about workplace visit

Diaries:

- Exercise diary
- Work diary

Other documents:

- Telephone interview registration form
- Workplace visit registration form
- Clinical shoulder evaluation manual
- How to use Axivity
- Informed consent

Danish versions of these documents can be requested from Jeanette Trøstrup (email: jeatro@rm.dk).
