# Supplementary material for: Reducing shoulder complaints in employees with high occupational shoulder exposures: study protocol for a cluster-randomised controlled study (The Shoulder-Café Study)
Source: Trials. 2019 Nov 12;20:627. doi: 10.1186/s13063-019-3703-y (PMC6852773; doi:10.1186/s13063-019-3703-y)
Supplement: Supplementary file 11 — Additional file 11: Table S2. Schedule for study procedures. For each batch of companies, the two interventions (Shoulder-Café and Shoulder-Guidance) start and end simultaneously. [file 13063_2019_3703_MOESM11_ESM.pdf]

Table 2: Schedule for study procedures. For each batch of companies, the two interventions (Shoulder-Café and Shoulder-Guidance) start and end simultaneously.

**Time point**	**Pre-intervention**	**Intervention**			**Follow-up**		
		**1st day (T_0)**	**Shortly after T_0**	**EOI 3 months after T_0**	**Shortly after EOI**	**6 months after T_0**	**12 months after T_0**
**ENROLMENT**							
Company randomisation *	x						
Informed consent	x						
Revealing randomisation result to participants		x **					
**INTERVENTION**							
Shoulder-Café		↔	↔	↔			
Shoulder-Guidance		↔	↔	↔			
**OUTCOME ASSESSMENTS**							
**Primary outcomes**							
**Hypothesis I**							
OSS at 6 month follow-up	x ***					x	
**Hypothesis II**							
Arm elevation > 60°			↔		↔		
**Secondary Outcomes**							
**Hypothesis I**							
OSS at 12 month follow-up	x ***						x
FABQ-PA at 6 month follow-up	x ***					x	
PGIC at 6 month follow-up						x	
FABQ-PA at 12 month	x ***						x

follow-up					
**Hypothesis II**					
Arm elevation > 90°		↔	↔		
Repetitive shoulder movements		↔	↔		
Forceful shoulder exertions		↔	↔		
Arm elevation > 30°		↔	↔		
**Supplementary outcomes**					
Hypothesis I					
NRS at rest	x			x	x
NRS during activity	x			x	x
Quick DASH + work module	x			x	
EQ5D-3L	x			x	
Work Ability Score	x			x	x
PGIC at 12 month follow-up					x
Satisfaction questionnaire				x	x
Felt informed about how to handle shoulder complaints, how to perform shoulder exercises, and how to reduce occupational shoulder exposures (questionnaire)				x	
**Hypothesis II**					
Work modification	x			x	
**OTHER ASSESSMENTS**					
Smoking status	x				
Body mass index	x				
Duration of shoulder complaints	x				
Psychosocial work exposures	x				

Occupational mechanical shoulder exposures (self-reported)	x				
Job title	x				x
Weekly working hours	x				x
System of wage payment	x				x
How often exercise was performed				x	x
Work status					x
Typical working day		↔	↔		
**ADHERENCE**					
Café meetings ****		⟷			
Exercise diary		⟷			
Work diary		↔	↔		
**CO INTERVENTIONS**					
Steroid injection				x	x
Shoulder surgery				x	x
Seen by doctor because of shoulder complaints				x	
Shoulder treatment by physiotherapist outside the project				x	
Shoulder treatment by chiropractor				x	
Pain medication in last 4 weeks	x			x	
**ADVERSE EVENTS**				x	

* Randomisation of a batch of companies takes place around 2 weeks before the start of an intervention. ** The randomisation result is revealed to the participants after the baseline questionnaire is filled in. *** The baseline questionnaire is scheduled to be filled in a few days before the 1st intervention day, but may be filled in on the 1st day (see **). **** For the intervention group, only.

Abbreviations: quick DASH = Disabilities of the Arm, Shoulder and Hand – quick version, EOI = end of intervention, EQ5D-3L = EuroQol 5D-3L, FABQ-PA = Fear Avoidance Beliefs Questionnaire – Physical Activity, NRS = numerical rating scale (range 0–10), OSS = Oxford Shoulder Score, PGIC = Patients' Global Impression of Change, T_0 = start of intervention.
